# L2Δ13, a splicing isoform of lysyl oxidase-like 2, causes adipose tissue loss via the gut microbiota and lipid metabolism

**DOI:** 10.1016/j.isci.2022.104894

**Published:** 2022-08-15

**Authors:** Yang Chen, Li-Xia He, Jin-Ling Chen, Xin Xu, Juan-Juan Wang, Xiu-Hui Zhan, Ji-Wei Jiao, Geng Dong, En-Min Li, Li-Yan Xu

**Affiliations:** 1 Guangdong Provincial Key Laboratory of Infectious Diseases and Molecular Immunopathology, Institute of Oncologic Pathology, Shantou University Medical College, Shantou 515041, Guangdong, P.R. China; 2 The Key Laboratory of Molecular Biology for High Cancer Incidence Coastal Chaoshan Area, Shantou University Medical College, Shantou 515041, Guangdong, P.R. China; 3 Department of Biochemistry and Molecular Biology, Shantou University Medical College, Shantou 515041, Guangdong, P.R. China; 4 Medical Informatics Research Center, Shantou University Medical College, Shantou 515041, Guangdong, P.R. China

**Keywords:** Lipid, Microbiome, Microbial metabolism

## Abstract

Obesity is primarily characterized by the dysregulation of lipid metabolism and gut microbiota. Here, we found that the body weight of transgenic mice overexpressing L2Δ13, a selectively spliced isoform of lysyl oxidase-like 2 (LOXL2), was lower than that of wild-type (WT) mice. Numerous microbiotas were significantly changed and most microbial metabolites were abnormal in L2Δ13 mice. Lipid metabolites in feces were negatively correlated with those in plasma, suggesting that L2Δ13 may affect lipid uptake, and potentially, adipose tissue homeostasis. This was supported by the weight loss and decreased area of adipose tissue in L2Δ13 mice. Adipogenic differentiation of primary stromal vascular fraction cells showed that the lipid droplets of L2Δ13 cells were significantly smaller than those of WT cells. Adipocyte differentiation-associated genes were also downregulated in adipose tissue from L2Δ13 mice. Thus, L2Δ13 can induce adipose tissue loss in mice by affecting gut microbiota homeostasis and multi-tissue lipid metabolism.

## Highlights

• L2Δ13 can affect gut microbiota homeostasis and bacterial metabolism
• L2Δ13 can disrupt lipid metabolism and cause weight loss in mice
• L2Δ13 inhibits adipocyte differentiation
• L2Δ13 may be a potential target for the treatment of obesity

## Graphical abstract

**Figure:**
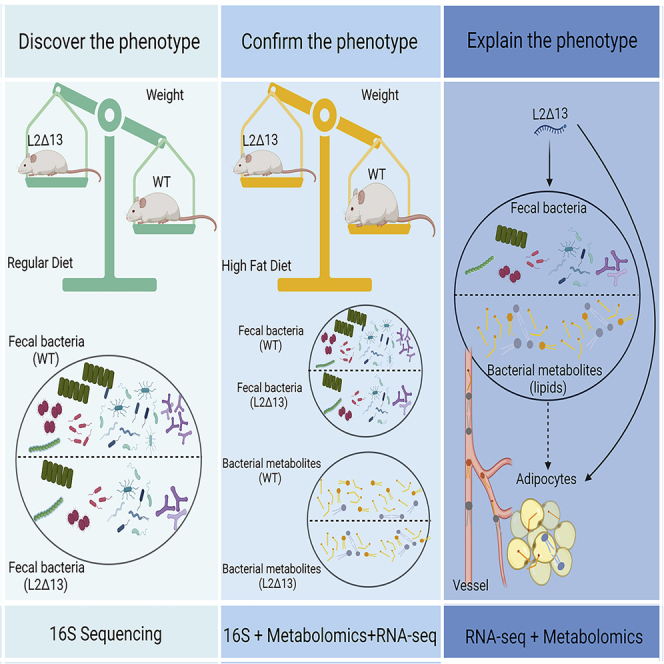

## Introduction

Interest in the relationship between gut bacteria and human health has surged in recent years. The gut microbiota is involved in several fundamental physiological functions that maintain metabolic homeostasis, including nutrient processing and digestion, metabolite production, and shaping of the immune system (Abdul Rahim et al., 2019; Lynch and Pedersen, 2016). The environment, particularly diet, has a substantial impact on the gut microbiota (Falony et al., 2016; Rothschild et al., 2018). Differences in food consumption have been shown to regulate the intestinal microbiota composition and its ability to store dietary energy, resulting in the development of various phenotypes (Caesar et al., 2015; Ridaura et al., 2013). A previous study fed mice an isocaloric diet containing either saturated or polyunsaturated fats and found that food intake in the saturated fat-fed mice increased, resulting in weight gain, obesity, increased inflammation, and metabolic abnormalities. In that study, the gut microbiome was found to differ between the two groups (Caesar et al., 2015). Advances in high-throughput sequencing may enable the microbiome to provide useful information for the study of microbiota and their potential functions (Pedersen et al., 2016). High-throughput data have linked differences in microbial communities to several diseases, including intestinal diseases, obesity, autoimmune diseases, and metabolic disorders (Clemente et al., 2012).

The incidence of obesity has increased sharply in the past forty years. Over the same period, the rate of obesity among males increased from 3% to 11%, while that among females increased from 6% to 15% worldwide (Jaacks et al., 2019). Today, obesity affects more than 650 million people globally (Albury et al., 2020). Obesity is a major risk factor for a variety of disorders, including liver disease, dementia, type 2 diabetes mellitus, and cancer (Ebrahimpour et al., 2020). Adipose tissue, particularly visceral adipose tissue, can act as an endocrine organ for the synthesis of obesity-related hormones and cytokines, which have been directly linked to diabetes and cancer risk (Avgerinos et al., 2019; Lopez-Suarez, 2019). The primary feature of obesity is impaired lipid metabolism, which can lead to the development of lipid metabolic disorders (Wang et al., 2014). Fatty acid production, fatty acid esterification, and other processes are involved in lipid metabolism in adipose tissue (Trayhurn and Wood, 2004). These disorders may lead to an increase in lipid levels, such as triglycerides and free fatty acids, which can cause abnormal lipid metabolism and accelerate the progression of metabolic syndrome (Trujillo and Scherer, 2006).

Members of the lysyl oxidase (LOX) family (LOX and LOXL1-4) play an important role in extracellular matrix (ECM) homeostasis and remodeling by catalyzing the final enzymatic steps required for the cross-linking of elastin and collagen (Wen et al., 2020). The ECM conveys signals that regulate lipid synthesis and accumulation (Romani et al., 2019). Recently, Watanabe et al. demonstrated that phospholipase A2 group V (sPLA2-V) can increase the expression of LOX (Watanabe et al., 2020). In fact, studies have confirmed the pathogenic role of LOX and LOXL2 in metabolic dysfunction, finding increased LOX mRNA levels in adipose tissue samples from high-fat diet models (Pang et al., 2021). Similarly, in a choline-deficient amino acid diet-induced non-alcoholic steatohepatitis (NASH) model, loss of LOXL1 in the liver ameliorated systemic lipid metabolism abnormalities, including normalizing weight loss, increasing serum triacylglycerol (TAG) levels by facilitating the transport of TAG from the liver to the bloodstream, and increasing fat weight (Dhariwal et al., 2017). These results suggest an association between LOX family members and lipid metabolism. The human LOXL2 gene, which is located on chromosome 8, encodes a protein containing 774 amino acids (Jourdan-Le Saux et al., 1998) and plays a critical role in various diseases and cancers. It regulates the tensile strength of extracellular tissue and affects several cellular processes, such as epithelial-mesenchymal transformation and cell differentiation (Peinado et al., 2005, 2008). Previously, we identified a novel LOXL2-selective splicing subtype lacking exon 13, referred to as L2Δ13, which encodes part of the conserved catalytic domain (Lv et al., 2014). L2Δ13 is mainly distributed in the cytoplasm and has the potential to influence cytoskeletal organization by interacting with intracellular actin-binding proteins (Zhan et al., 2019). L2Δ13 may also enhance the invasiveness and metastasis of cancer cells (Zhan et al., 2019). In this study, we used a transgenic mouse model overexpressing L2Δ13 and found that the body weight and adipose tissue of the mice were lower than those of wild-type mice during rearing. Multi-dimensional omics were used to analyze the function of L2Δ13 in microbial homeostasis, lipid metabolism, and adipose loss.

## Results

### L2Δ13 affects bacterial homeostasis and decreases mouse body weight

**Figure 1:**
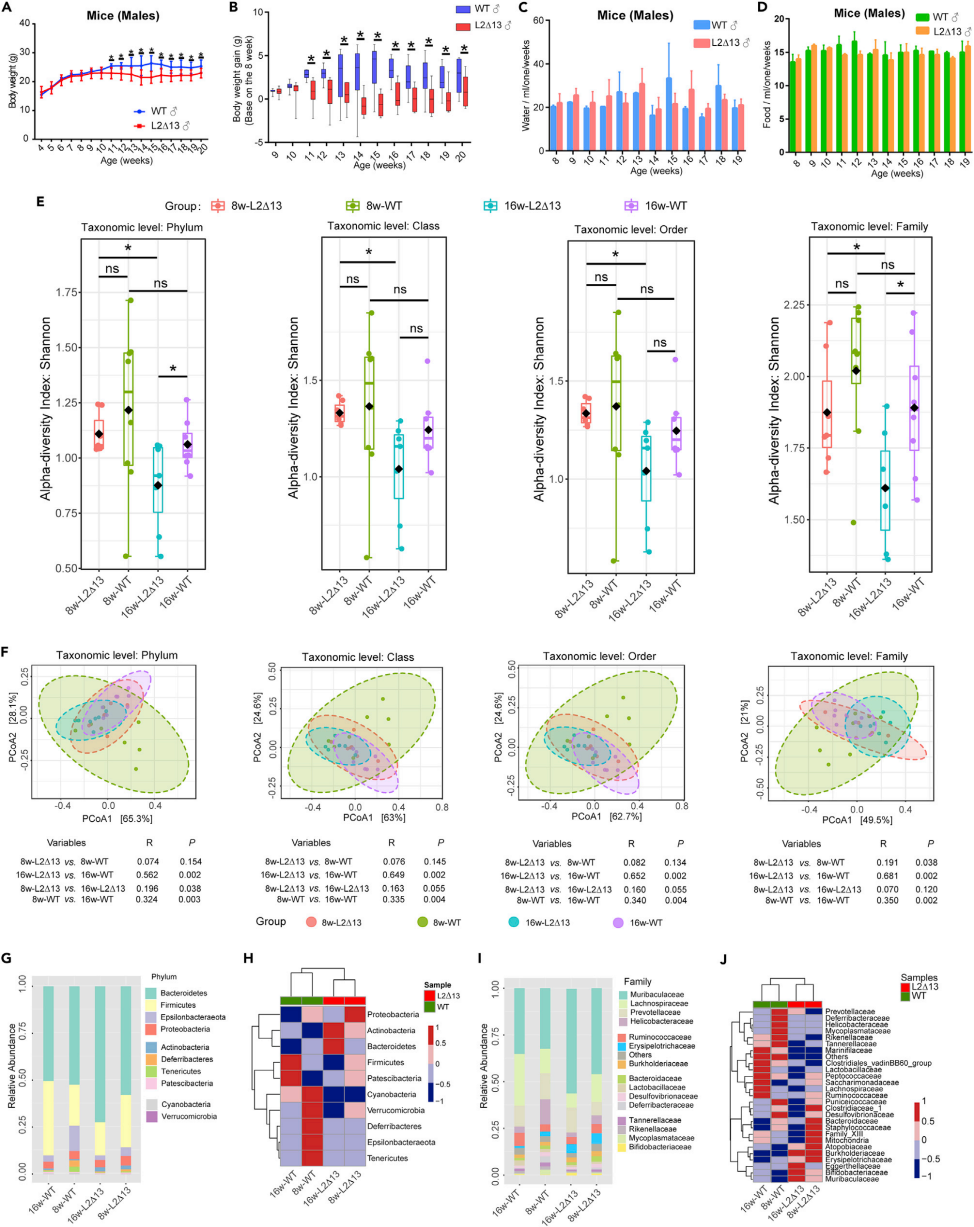
L2Δ13 mice display abnormal bacterial homeostasis and reduced body weight when fed a normal diet (A) Weight monitoring of L2Δ13 and WT mice. From week eleven, the body weight of L2Δ13 mice was significantly lower than that of wild-type mice. (B) Body weight gain in both groups. The weight at week eight was selected as the baseline. The weight gain of L2Δ13 mice was significantly lower than that of WT mice. (C) Water monitoring of L2Δ13 and WT mice. There was no significant difference in water intake between the two groups. (D) Food monitoring of L2Δ13 and WT mice. There was no difference in dietary intake between the two groups. (E) Alpha diversity of the fecal microbiome in L2Δ13 and WT mice. The Shannon index was used. (F) Beta diversity of the fecal microbiome. Each sample is colored based on its group. (G and I) The overall composition of the microbiota at the phylum (G) and family (I) levels in L2Δ13 and WT mice at each time point. (H and J) Cluster analysis of the microbiota at the phylum (H) and family (J) levels in the two groups. (∗p < 0.05; ∗∗p < 0.01; ∗∗∗p < 0.001; L2Δ13 vs wild-type mice).

**Figure 2:**
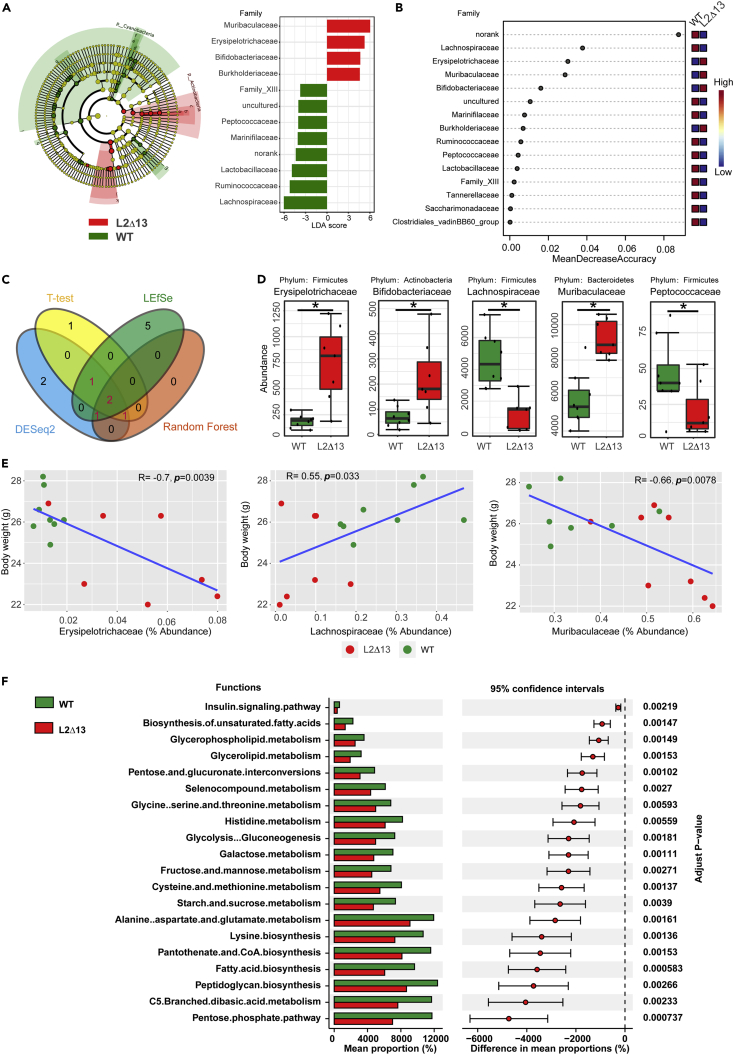
Multiple methods used to screen predominant microbiota (A) LEfSe analysis. Histogram of LDA scores showing the features with differential abundance in L2Δ13 and WT mice. (B) Random forest analysis. Dot plot showing the top metabolites ranked by the mean decreased accuracy. (C) Venn plot showing the predominant bacterial species that were selected by four common methods (t-test, DESeq2, LEfSe, and random forest). Red denotes predominant bacterial species, which were stable and different in at least three algorithms. (D) The abundance of five identified crucial phyla. (E) Three out of the five crucial phyla were related to body weight. (F) PICRUSt2 results revealed the potential function of the microbiota affected by L2Δ13. (∗p < 0.05; ∗∗p < 0.01; ∗∗∗p < 0.001; L2Δ13 vs wild-type mice).

In the male group, after week 11 (Figure 1A), the body weights of the L2Δ13 mice were lower than those of the WT mice (p < 0.05, Figure 1B). The change in body weight in the L2Δ13 mice was independent of the amount of food and water consumed by the mice (Figures 1C and 1D). Changes in body weight are directly related to alterations in the gut microbiota (Lynch and Pedersen, 2016). Thus, 16S rRNA sequencing was performed to examine the fecal microbiota profiles of L2Δ13 and WT mice. Based on the microbiota composition of L2Δ13 and WT mice, we found that the diversity of phyla and families differed significantly at week 16, while there were no differences in other taxonomic levels (Figure 1E). Based on beta diversity at week 16, the WT and L2Δ13 mice could be classified into two distinct groups at the phylum and family levels (R_Phylum_ = 0.562, *P*_Phylum_ = 0.002; R_Family_ = 0.681, *P*_Family_ = 0.002), indicating that the gut microbial population differed between the two groups (Figure 1F). At the phylum level, *Bacteroidetes* and *Firmicutes* were the dominant phyla in the L2Δ13 and WT mice (Figure 1G). L2Δ13 enhanced the abundance of *Bacteroidetes* and decreased the abundance *of Firmicutes* (Figure 1H). Muribaculaceae, Lachnospiraceae, and Prevotellaceae were the dominant families (Figure 1I). L2Δ13 attenuated the abundance of Lachnospiraceae and Ruminococcaceae and increased the abundance of Muribaculaceae, Erysipelotrichaceae, and Burkholderiaceae at week 16 (Figure 1J). The microbiota of L2Δ13 mice was significantly different from that of the WT mice. Muribaculaceae, Erysipelotrichaceae, Bifidobacteriaceae, and Burkholderiaceae were enriched in L2Δ13 mice, whereas Lachnospiraceae, Ruminococcaceae, Lactobacillaceae, and Peptococcaceae were enriched in WT mice (Figure 2A). The greatest differences were found between the Muribaculaceae, Erysipelotrichaceae, Bifidobacteriaceae, and Lactobacillaceae families (Figure 2B). Next, we integrated the results of the four differential algorithms and filtered five critical microbiotas with stable and different results in at least three algorithms (Figure 2C). Muribaculaceae, Erysipelotrichaceae, and Bifidobacteriaceae were more abundant in L2Δ13 mice than in WT mice, while Lachnospiraceae and Peptococcaceae were less abundant (Figure 2D). Subsequently, we found that the abundances of Muribaculaceae, Erysipelotrichaceae, and Lachnospiraceae were related to body weight (Figure 2E). The results of PICRUSt2 suggested that the change in microbiota induced by L2Δ13 was closely related to metabolism (adjusted p < 0.05), particularly, the metabolic processes of fatty acid biosynthesis and glycerophospholipid metabolism (Figure 2F).

We also analyzed the microbiome of female mice. The body weight of female mice was still lower in the L2Δ13 mice compared with WT mice (Figure S3A). However, the microbiomes showed no differences (Figures S3B and S3C). Similarly, under HFD, WT female mice were still heavier than L2Δ13 mice (Figure S4A). Similarly, the microbiomes showed no differences (Figures S4B and S4C). In short, both male and female L2Δ13 mice are lighter in body weight. However, only the male mouse microbiome is changed. There may be the estrogen protection in female mice and then bias the function of L2Δ13. In the other hand, some studies have found sex differences in obesity and some metabolic diseases (Chella Krishnan et al., 2021; Gao et al., 2021). To avoid the sex influence, we only analyzed male mice in the subsequent analyses.

### L2Δ13 alters bacterial homeostasis and decreases body weight gain in mice fed a high-fat diet

**Figure 3:**
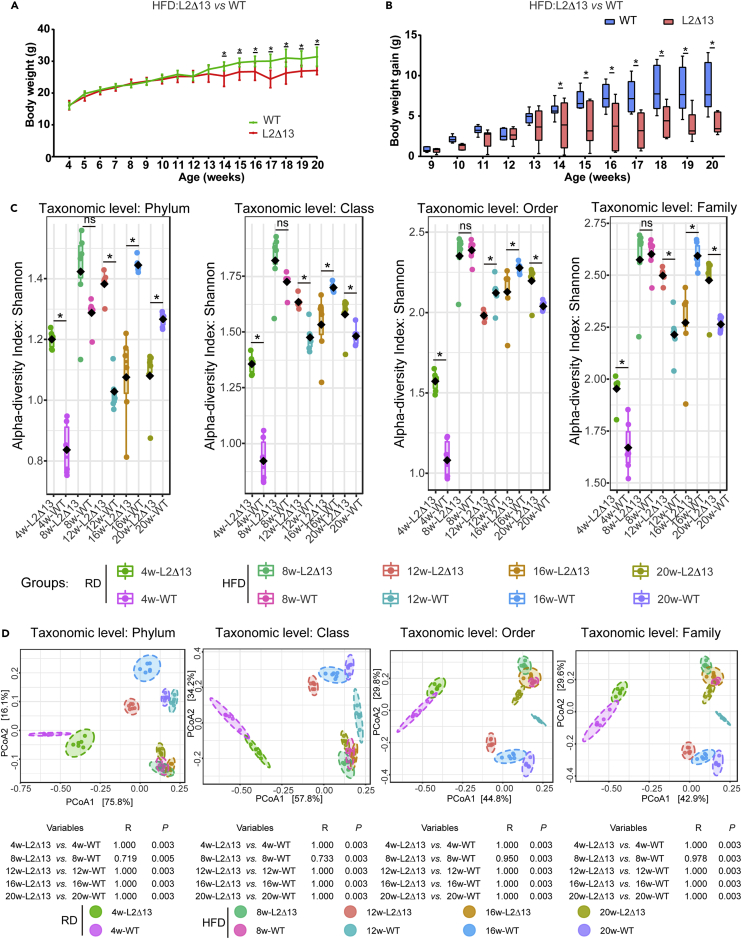
Under a high-fat diet, the bacterial homeostasis of L2Δ13 mice was altered and the weight gain was less than in WT mice (A) Weight monitoring of L2Δ13 and WT mice. From week 14, the body weight of L2Δ13 mice was significantly lower than that of WT mice. (B) Body weight gain in the two groups. Weight at week 8 was selected as the baseline. The weight gain of L2Δ13 mice was significantly lower than that of WT mice. (C) Alpha diversity of the fecal microbiome in L2Δ13 and WT mice. The Shannon index was used. ∗ indicates a p value < 0.05. (D) Beta diversity of the fecal microbiome. Each sample is colored according to its group.

**Figure 4:**
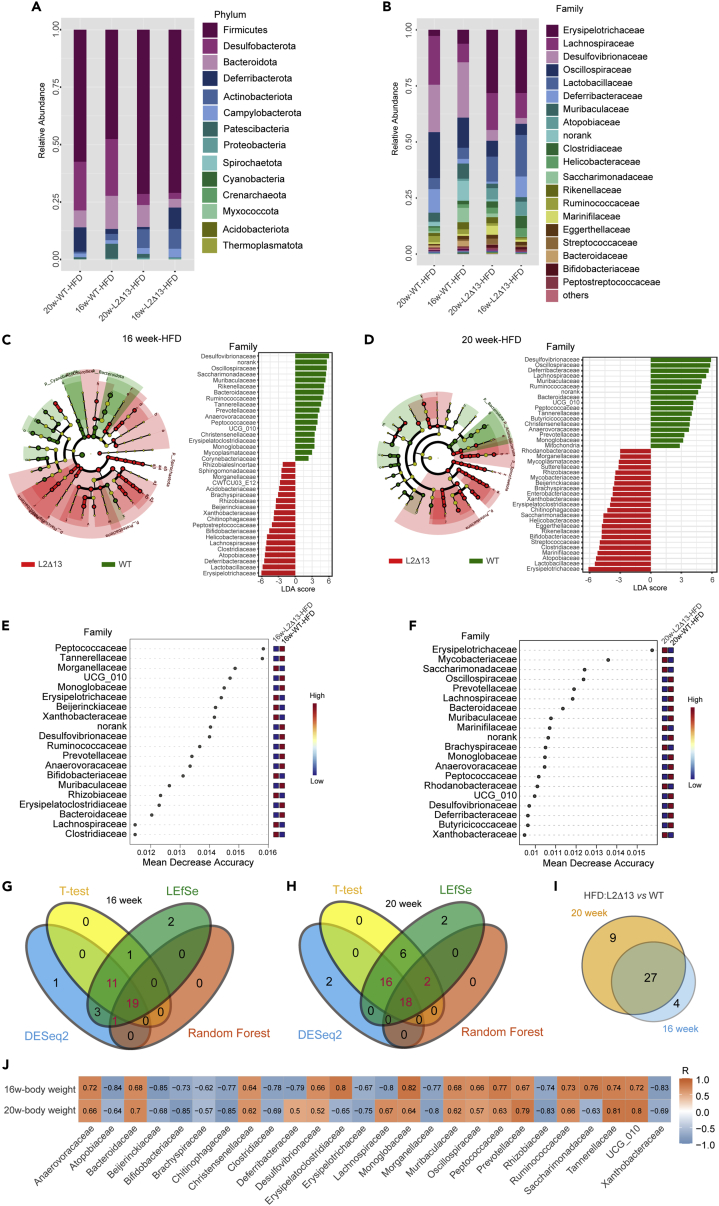
The predominant microbiota components in L2Δ13 and WT mice fed a high-fat diet (A and B) The overall composition of the microbiota at the phylum (A) and family (B) levels in L2Δ13 and WT mice at weeks 16 and 20. (C and D) LEfSe analysis at weeks 16 (C) and 20 (D). (E and F) Random forest analysis at 16 (E) and 20 (F) weeks. (G and H) Venn plot showing the predominant microbiota filtered by the four common methods at 16 (G) and 20 (H) weeks. Red denotes the predominant microbiotas which were stable and different in at least three algorithms. (I) Twenty-seven common crucial microbiota were identified at week 16. (J) 22 out of the 27 crucial microbiotas were related to body weight. (∗p < 0.05; ∗∗p < 0.01; ∗∗∗p < 0.001; L2Δ13 vs wild-type mice).

Next, we constructed a male HFD mouse model to validate the role of L2Δ13 in mice. The L2Δ13 mice weighed less than the WT mice from week 14 (Figure 3A), and the weight gain remained lower after this time point (Figure 3B). The HFD exacerbated differences in the microbiota, and even after significant changes in body weight, the alpha diversity of L2Δ13 mice remained significantly different from that of the WT mice at all taxonomic levels (Figure 3C). In weeks 16 and 20, the beta diversity of the gut microbiota of WT and L2Δ13 mice could be classified into two distinct groups at the phylum and family levels (Figure 3D). The gut microbiota of L2Δ13 mice was distinct from that of the WT mice. Comparing the overall community composition of L2Δ13 and WT mice, *Bacteroidetes*, *Firmicutes,* and *Desulfobacterota* were the most dominant phyla in L2Δ13 and WT mice after feeding an HFD (Figure 4A). The most dominant families were Erysipelotrichaceae, Lachnospiraceae, Desulfovibrionaceae, Oscillospiraceae, Lactobacillaceae, and Deferribacteraceae (Figure 4B). Numerous distinct microbial species were observed among the L2Δ13 and WT mice after being fed an HFD. Erysipelotrichaceae, Lactobacillaceae, and Atopobiaceae were enriched in L2Δ13 mice, while Desulfovibrionaceae, Oscillospiraceae, and Muribaculaceae were enriched in WT mice (Figures 4C and 4D). Muribaculaceae, Erysipelotrichaceae, and Peptococcaceae were the most predominant families (Figures 4E and 4F). Integration of the results from the four algorithms revealed that 31 critical microbial species differed at week 16, and 36 differed at week 20 (Figures 4G and 4H). At both time points, 27 of these critical microbial species were stable and different (Figure 4I). Next, we identified 26 microbial species that were related to body weight, with 22 displaying the same tendency in both weeks 16 and 20 (Figure 4J). The PICRUSt2 results also indicated that the microbiota altered by L2Δ13 were closely associated with metabolism, particularly fatty acid metabolism and glycerophospholipid metabolism (Figure S5). These data indicate that L2Δ13 influences gut microbiota and prevents body weight gain in mice.

### L2Δ13 influences the lipid metabolites of microbiota and plasma in mice

**Figure 5:**
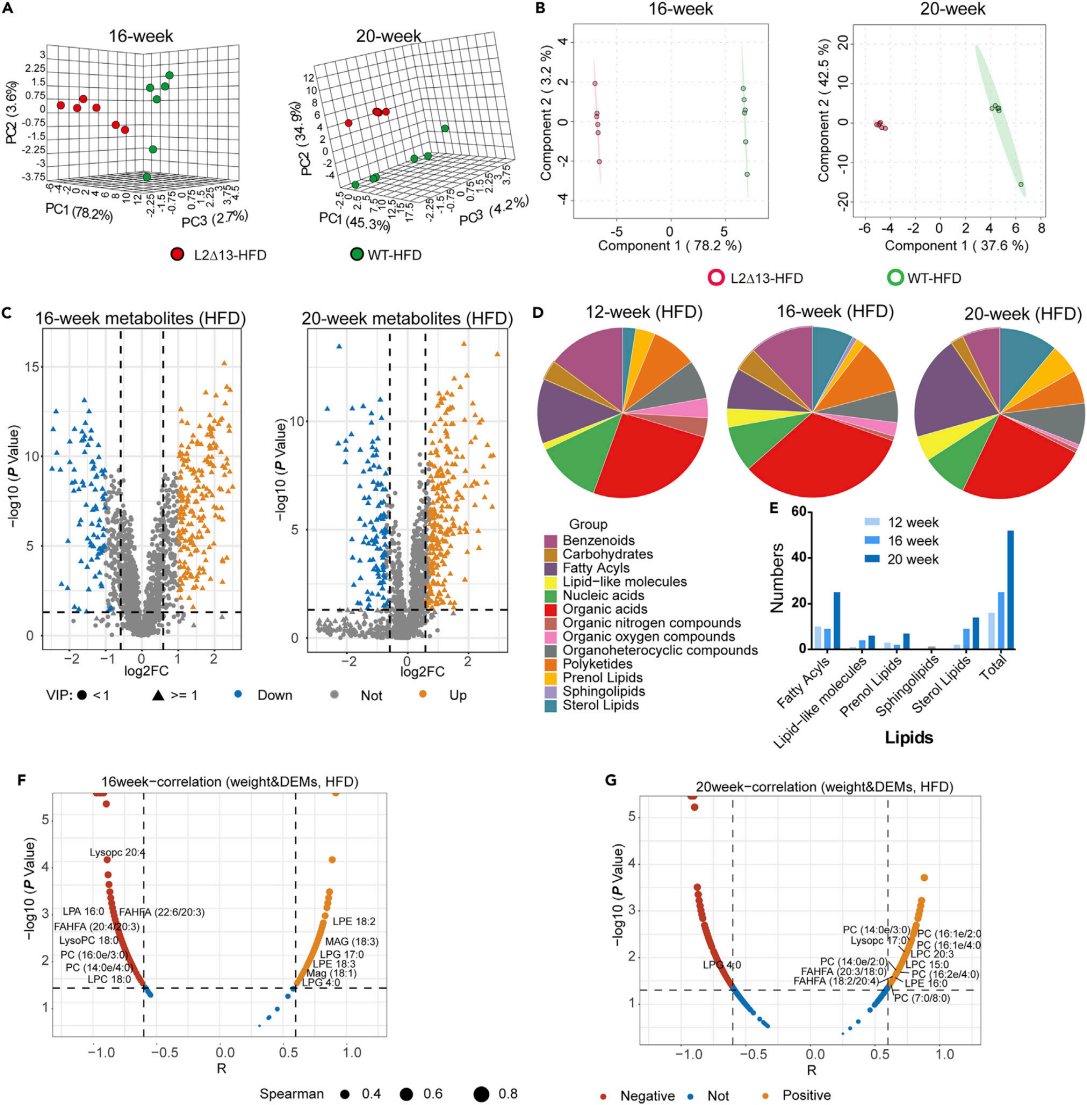
Microbial metabolome analysis of L2Δ13 and WT mice (A) PCA analysis of the two groups at weeks 16 and 20. (B) PLS-DA was used to cluster the fecal metabolites. (C) Volcano plot showing the differentially expressed metabolites (DEMs) in L2Δ13 and WT mice at weeks 16 and 20. (D) Pie plot showing the subclass of DEMs before (12 weeks) and after (16 and 20 weeks) a significant change in weight. (E) Histogram showing the number of differentially expressed lipids at these time points, showing that lipids increased before and after weight change. (F and G) The DEMs were related to body weight at weeks 16 (F) and 20 (G).

Non-targeted metabolomics analysis was performed to determine the microbial metabolites in L2Δ13 and WT mice, and to elucidate the relationship between the gut microbiome and metabolites. PCA and PLS-DA revealed a clear separation between WT and L2Δ13 mice. It also revealed that there were significant differences in microbial metabolites between the two groups (Figures 5A and 5B). As shown in Figure 5C, there were 364 differentially expressed metabolites (DEMs) at week 16 and 372 DEMs at week 20 between L2Δ13 and WT mice (adjusted p < 0.05, |log2fold-change| >0.585 and VIP ≥1). We then summarized the categories of DEMs before and after the change in body weight (Figure 5D). As the difference in body weight gradually increased, so did the difference in lipids (Figure 5E). In addition, L2Δ13 was found to influence body weight by affecting lipid metabolism. The most differentially expressed lipid metabolites, including PC (14:0e/3:0), LPE 18:2, and FAHFA (20:4/20:3), correlated with body weight (Figures 5F and 5G). Most DEMs were correlated with the gut microbiota (Figure S6). Overall, these results indicate that L2Δ13 has the potential to influence body weight by affecting the gut microbiota and associated lipid metabolites.

**Figure 6:**
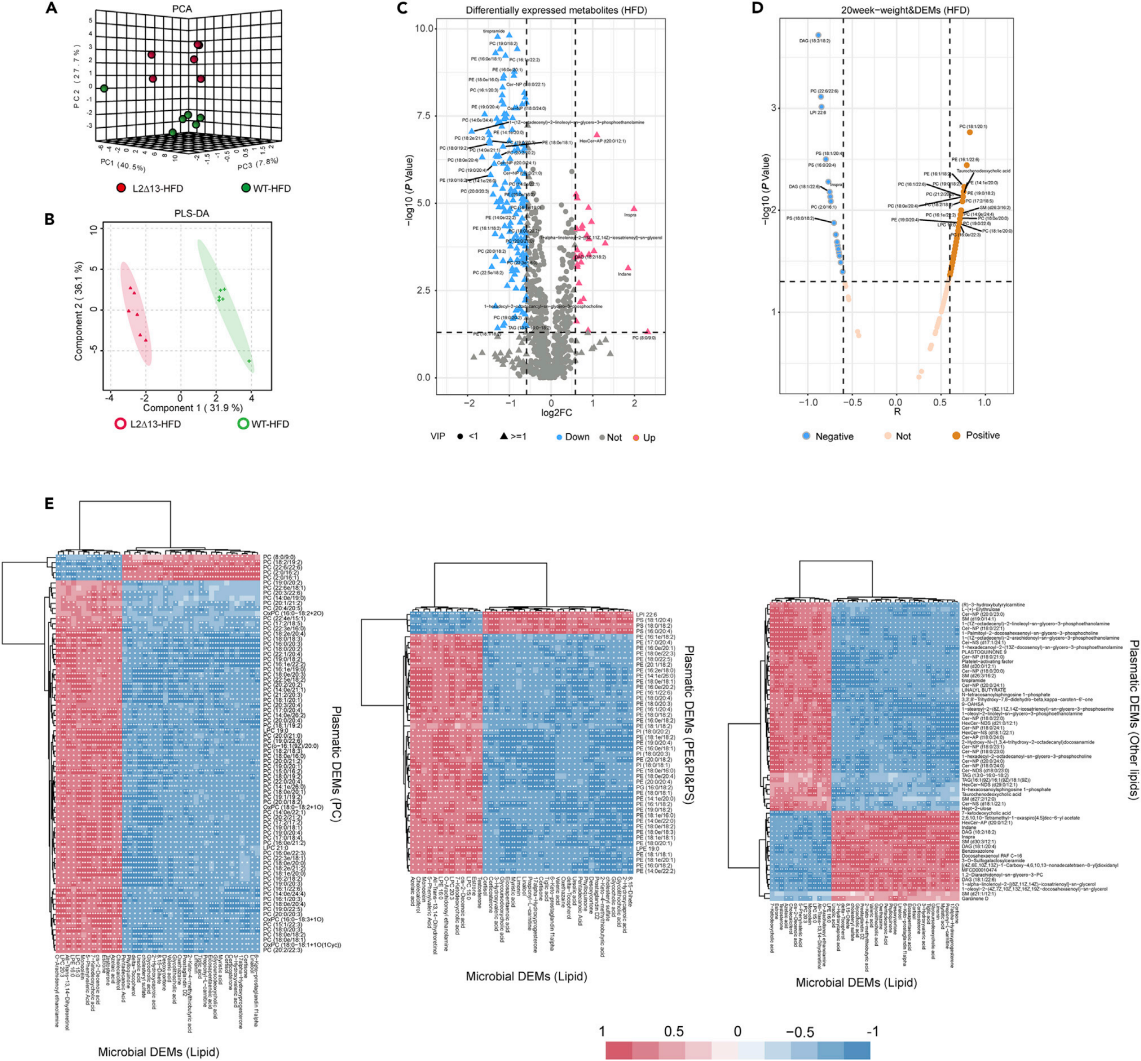
Plasmatic metabolome analysis in L2Δ13 and WT mice (A) PCA analysis of the two groups. (B) PLS-DA was used to cluster the plasma metabolites. (C) Volcano plot showing the differentially expressed metabolites (DEMs) in the plasma of L2Δ13 and WT mice. (D) Plasmatic DEMs were related to body weight. (E) Plasmatic DEMs were significantly related to microbial DEMs. (∗p < 0.05; ∗∗p < 0.01; ∗∗∗p < 0.001; L2Δ13 vs wild-type mice).

At the end of week 20, lipid metabolites were determined in plasma (Figure 6). There was a clear separation between WT and L2Δ13 mice (Figures 6A and 6B). This suggested that the lipid metabolites in plasma differed between the two groups. There were 160 downregulated DEMs and 28 upregulated DEMs (adjusted p < 0.05, |log2fold-change| >0.585, and VIP ≥1; Figure 6C). Subsequently, we found that most of the DEMs were related to changes in body weight (Figure 6D), and there were also significant relationships with the DEMs of microbiota and plasma (Figure 6E). Interestingly, most of the differentially expressed lipids in feces were upregulated, and most of the differentially expressed lipids in plasma were downregulated, suggesting that L2Δ13 may inhibit the absorption of lipids from the intestine to the plasma (Table S3). In summary, these results suggest that metabolites affected by L2Δ13 can influence other tissues or organs via systemic circulation.

### L2Δ13 reduces adipose tissue weight and induces abnormal lipid metabolism in mice

**Figure 7:**
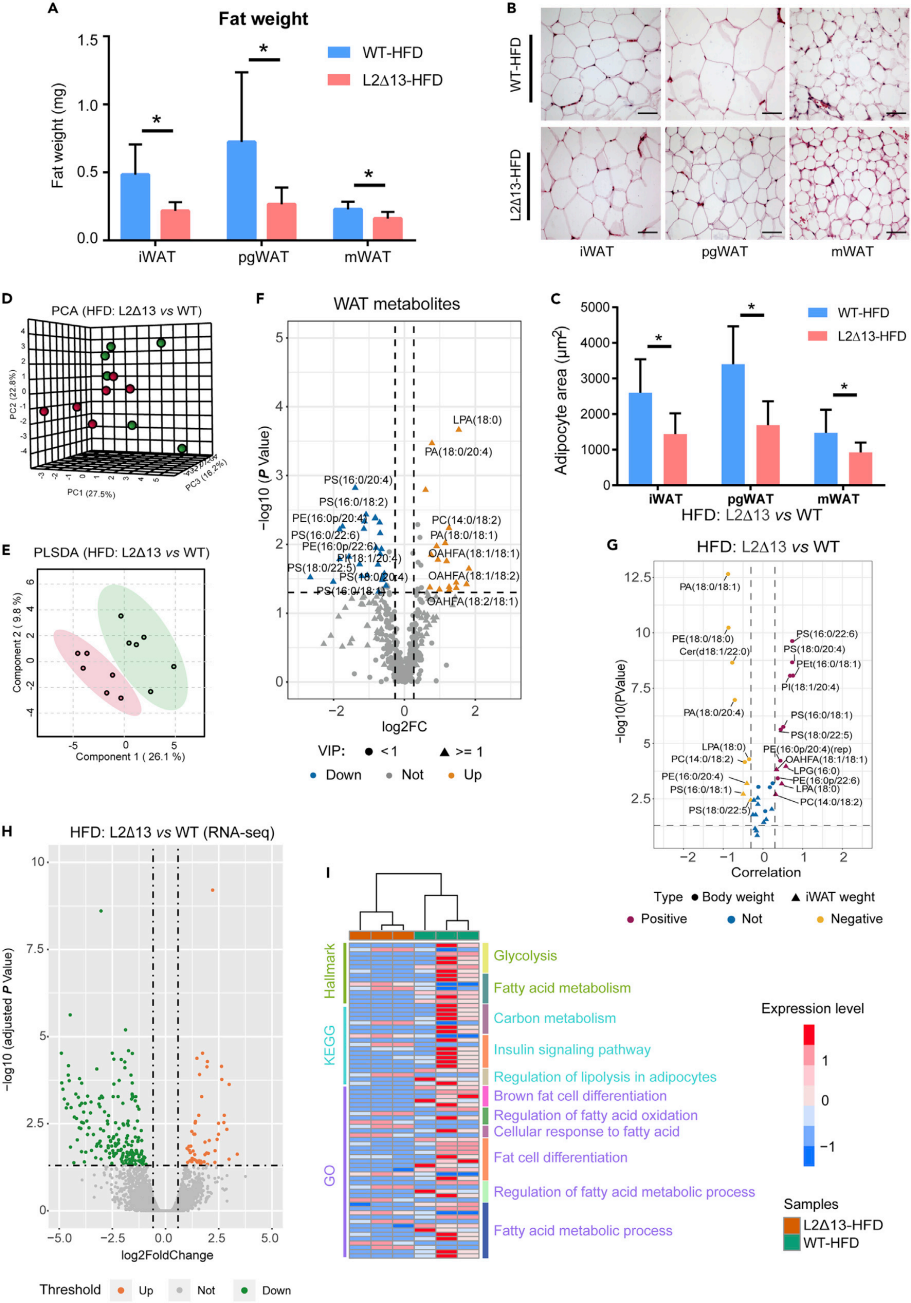
L2Δ13 affects lipid metabolism in adipose tissue (A) WAT in L2Δ13 mice was significantly lower than that in WT mice. (B) HE staining shows the area of WAT per unit field of vision. Scale bars represent 50 μm. (C) Statistical analysis of HE showing that the area of white adipocytes in L2Δ13 mice was significantly smaller than that in WT mice. (D) PCA analysis of the WAT metabolome. (E) PLS-DA shows clusters of WAT metabolites in the two groups. (F) Volcano plot showing DEMs in WAT. (G) DEMs were related to body weight and WAT weight. (H) Volcano plot showing differentially expressed genes (DEGs) in WAT. (I) Enrichment analysis showed that the DEGs were related to fatty acid metabolism and fat cell differentiation. (∗p < 0.05; ∗∗p < 0.01; ∗∗∗p < 0.001; L2Δ13 vs wild-type mice).

Body weight and fat accumulation are inextricably linked. To determine whether variations in weight in L2Δ13 mice were caused by alterations in adipose tissue, the amounts of fat from the L2Δ13 and WT mice were compared. The white adipose tissue (WAT) weight in L2Δ13 mice was lower than that in WT mice (Figure 7A). Next, HE staining, to determine the WAT area per unit field of vision (Figure 7B), showed the WAT area in L2Δ13 mice was significantly less than that in WT mice (Figure 7C). Subsequently, lipid metabolomics was used to determine the lipid metabolites in WAT. The WAT lipid metabolites in L2Δ13 mice differed from those in WT mice (Figures 7D–7F). Differentially expressed lipid metabolites were associated with body weight and WAT weight (Figure 7G). RNA-seq of adipose tissue was performed to investigate the mechanism by which L2Δ13 influences adipose tissue differentiation. Overall, 55 upregulated and 338 downregulated differentially expressed genes (DEGs) were identified between L2Δ13 and WT mice fed a high-fat diet (Figure 7H). Enrichment analysis revealed that these DEGs were also enriched in lipid metabolism and fat cell differentiation (Figure 7I and Table S4).

### L2Δ13 inhibits adipose tissue differentiation and induces adipose tissue loss

**Figure 8:**
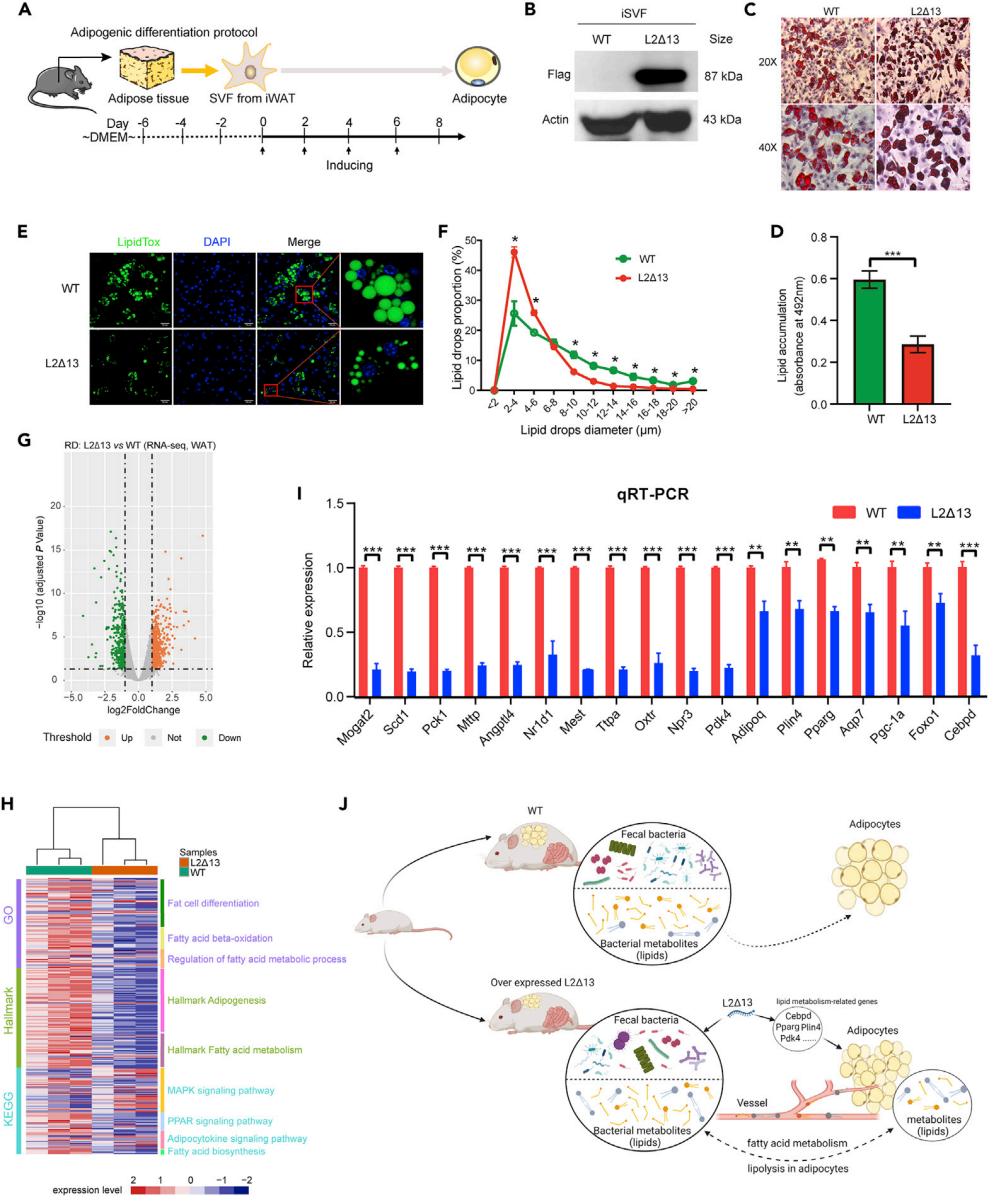
L2Δ13 restricts adipocyte differentiation and inhibits the expression of genes related to adipogenesis and adipocyte differentiation (A) Schematic diagram of the isolation and adipogenic differentiation of primary adipose stem cell SVF *in vitro*. (B) Expression of L2Δ13 in primary cell SVF was detected by western blotting. β-Actin was used as an internal reference protein control. (C and D) Differentiated adipocytes were detected by Oil Red O staining. Nuclei were stained with hematoxylin, and red staining showed the presence of neutral lipids. Scale bars represent 50 μm. (E and F) Detection of lipid droplets after differentiation with neutral lipid droplet staining. Lipid droplets were stained with LipidTOX, and the nuclei were stained with DAPI. Magnification:×200 and ×400. Scale bars represent 50 μm. There were three mice per group. (G) Volcano plot showing DEGs in WAT. (H) Enrichment analysis showed that the DEGs were related to fatty acid metabolism and fat cell differentiation. (I) mRNA levels of DEGs related to adipogenesis were evaluated by qRT-PCR. There were three mice per group. (J) The diagram shows the mechanism by which L2Δ13 inhibits obesity, created with BioRender.com. (∗p < 0.05; ∗∗p < 0.01; ∗∗∗p < 0.001; L2Δ13 vs wild-type mice).

To investigate whether L2Δ13 could directly inhibit adipocyte differentiation, an adipose-derived stem cell SVF from L2Δ13 mice was constructed *in vitro* (Figures 8A, 8B, and S7). Oil red O staining was performed after 8 days of differentiation. Lipid accumulation in adipocytes from L2Δ13 mice was reduced compared to that in adipocytes from WT mice, and lipid droplets in the cytoplasm were relatively small (Figures 8C and 8D). Neutral lipid droplet staining also confirmed that there were fewer and smaller lipid droplets in adipocytes from L2Δ13 mice (Figure 8E), which is consistent with the results from the oil red O staining. Adipocytes from L2Δ13 mice contained more lipid droplets of 2–4 μm in diameter, fewer lipid droplets of 8–14 μm in diameter, and very few lipid droplets of more than 16 μm in diameter, compared to WT mice (Figure 8F). These results suggest that L2Δ13 may inhibit adipocyte differentiation.

### L2Δ13 inhibits adipose tissue differentiation-related genes

To confirm whether L2Δ13 could affect lipid metabolism and influence adipose tissue differentiation, we analyzed the transcriptome of adipose tissues used to obtain SVF cells (Figure 8G). Enrichment analysis revealed that the DEGs were also enriched in lipid metabolism and fat cell differentiation (Figure 8H and Table S5). The validated qRT-PCR data were comparable to the RNA-seq data. The expression of adipogenic-related genes, including *Scd1, Aqp7, Fabp4, Angptl4, Nr1d1, AdipoQ,* and *Plin4*, was downregulated (Figure 8I). In summary, these findings suggest that L2Δ13 inhibits the expression of adipogenesis-related genes and reduces adipocyte differentiation.

## Discussion

In this study, we elucidated the mechanism underlying L2Δ13-induced weight loss in mice via microbiome, metabolome, and transcriptome analyses. The main findings can be summarized as follows. The gut microbiota and metabolism are stable in WT mice, and adipose tissue differentiates normally. When L2Δ13 is overexpressed, bacterial homeostasis is disrupted, bacterial metabolites and plasmatic metabolic homeostasis are changed. Subsequently, L2Δ13 also suppresses the expression of genes related to adipose differentiation, resulting in limited adipose differentiation and lower body weight in mice, and the metabolic homeostasis is disrupted in adipose tissue via related metabolic pathways, including fatty acid metabolism and lipid synthesis in adipose tissue (Figure 8J). This suggests that direct metabolic regulation of lipid metabolism in adipocytes by L2Δ13 occurs, by cytoskeletal and transcription factor regulation associated with L2Δ13, which secondarily causes low fat and weight.

Over the past decade, the potential role of gut microbiota in human disease has received widespread attention. Several studies have reported that the gut microbiota plays an important role in metabolic disorders, such as obesity and type 2 diabetes mellitus (Forslund et al., 2015; Turnbaugh et al., 2006). Gut microbiota convert food into nutrients and play an important role in host energy intake. The gut microbiota associated with obesity can utilize energy from the diet (Turnbaugh et al., 2006). Previous studies have reported that obese individuals present with significant differences in the abundance of microbes, such as *Oscillibacter* (Kim et al., 2019), Clostridiaceae (Forslund et al., 2015), and Erysipelotrichaceae (Nolan et al., 2017) compared with non-obese individuals. In this study, the microbiota was significantly changed under the influence of L2Δ13, suggesting that L2Δ13 may play a crucial role in obesity. Gut microbiota produce key compounds, including short-chain fatty acids, bile acids, phenols, and ammonia. These metabolites are involved in communication between microbiota and the host and are important for mediating host physiology (Schroeder and Backhed, 2016). Metabolomic studies based on the gut microbiome have provided useful insights into metabolic diseases (Chen et al., 2019).

Several omics approaches have been developed to explore the function of microbiome-derived metabolites, including metagenomics and metabolomics. These methods are used to explore host metabolism, quantify compounds of interest, and explore the relationship between the gut microbiome and the host (Vernocchi et al., 2016). Several fecal or blood metabolites are associated with metabolic disorders (Monnerie et al., 2020). In the current study, we found that L2Δ13 is highly correlated with abnormal bacterial homeostasis and metabolism, which may be implicated in the pathogenesis of metabolic disorders. However, the exact mechanism by which L2Δ13 affects the microbiota and results in adipose loss remains unclear. In recent years, fecal microbiota transplantation has been investigated widely in various diseases (Kaakoush, 2020; van den Berg et al., 2021). Therefore, further studies on fecal microbiota transplantation in our mouse model will help to clarify this mechanism. In addition, our results also showed that there was no significant difference in the microbiota in female mice, suggesting sex differences in the disease mechanisms regulated by L2Δ13. The study of women in biology research has been vacant for too long, and it has only been in the last few years that attention has been gradually drawn to it. Women as research subjects began to be more systematically included as research participants in the 1990s (Dhariwal et al., 2017). Women and men differ in prevalence, risk, presentation, disease physiology, and response to clinical interventions (Dhariwal et al., 2017). Recently, the importance of sex differences to the understanding of human health and disease has been recognized, and the latest research has raised the importance of incorporating sex differences into basic scientific research (Pang et al., 2021). Studies have found that fat distribution and obesity in adults may also contribute to sex-dependent microbiological differences (Min et al., 2019). Several animal studies also found significant differences in gut microbiota composition by sex in mice (Elderman et al., 2018; Org et al., 2016). For example, the Bacteroides and Lactobacillus in B_6_ female mice are more common than these in B_6_ males and in BALB/c male mice; Bifidobacterium is less than BALB/c females (Elderman et al., 2018). Here, we will continue to explore the sex differences in L2Δ13 mice in the future.

LOXL2 contains four scavenger receptor cysteine-rich (SRCR) domains, which possess deacetylase activity and a C-terminal lysyl oxidase domain (Ma et al., 2017; Wen et al., 2020). As a splice variant of LOXL2, L2Δ13 also contains SRCR domains, although deamination activity is impaired (Wen et al., 2020). Scavenger receptors are membrane proteins with a unique structure and were initially recognized and classified based on their ability to bind to modified low-density lipoprotein (Goldstein et al., 1979; Parthasarathy et al., 1986). Studies have reported that scavenger receptors mediate the uptake of cholesterol esters and the bidirectional flow of free cholesterol, which are essential for lipoprotein metabolism (Connelly et al., 2001). Hepatic scavenger receptors selectively absorb high-density lipoprotein cholesterol esters (HDL-CE) to transport cholesterol to bile, thus maintaining HDL function, and play an important role in the removal of residual lipoproteins (Linton et al., 2017; Shen et al., 2018). Some scavenger receptors, such as CD36 (also known as scavenger receptor B2), bind long-chain fatty acids, phospholipids, and oxidized lipids, and are thus implicated in lipid accumulation and inflammatory responses (Yang et al., 2017). A previous study by our team identified several L2Δ13-binding cytoskeletal proteins, including ezrin and fascin (Zhan et al., 2019). Cytoskeletal proteins are regulated by phospholipids, mainly phosphatidylinositol (Anderson and Marchesi, 1985). Phosphatidylinositol plays a key role in signal transduction as a precursor of second messengers and scaffold-targeting molecules (Yin and Janmey, 2003). Here, by exploring the mechanism of L2Δ13-driven adipose loss through the metabolome, we also found that a group of phospholipids displayed metabolic abnormalities under the effect of L2Δ13. These abnormal phospholipids are highly correlated with body weight and WAT weight in mice, suggesting that L2Δ13-medicated metabolic disorders may be associated with its SRCR domains.

Studies have explored the relationship between adipocyte differentiation, obesity, and adipose loss (Derecka et al., 2012; Hammarstedt et al., 2018; Mauro et al., 2017). In this study, we evaluated the expression levels of several genes related to adipocyte differentiation and metabolism and found that they are inhibited by L2Δ13. For example, *Scd1* and *Nr1d1* are crucial for lipid metabolism (Iizuka et al., 2020; Yang et al., 2015), *Angptl4* regulates lipid metabolism and influences the activity of lipoprotein lipase (Kristensen et al., 2021), and *Plin4* is associated with the development of lipid droplets (Gimenez-Andres et al., 2021). These findings provide a basis for further exploring the regulatory mechanism of L2Δ13 in obesity. Nevertheless, the role of L2Δ13 in the overall regulatory network of lipid metabolism remains unclear; further studies are needed to investigate whether it interacts directly or indirectly with lipid metabolism-associated enzymes. In addition, since L2Δ13 leads to adipose loss, enhancing LOXL2 gene splicing to form L2Δ13 may represent a potential strategy for treating obesity. Consequently, the splicing regulators that splice LOXL2 into L2Δ13 need to be identified.

In conclusion, L2Δ13, a spliced LOXL2 isoform, affects gut microbiota homeostasis and bacterial metabolism, and participates in lipid metabolic processes. It can also decrease the expression of adipocyte differentiation-related genes and inhibit adipocyte differentiation. Thus, L2Δ13 may be a potential target gene for the treatment of obesity.

### Limitations of the study

The exact mechanism by which L2Δ13 affects the microbiota and results in adipose loss remains unclear. In recent years, fecal microbiota transplantation (FMT) has been investigated widely in various diseases (Kaakoush, 2020; van den Berg et al., 2021). Therefore, further studies on FMT in our mouse model will help to clarify this mechanism.

## Data availability statement

• Raw data of this study are available from the corresponding author L.Y.X. on request.
• This paper does not report any original code.
• The derived data supporting the findings of this study are available in GitHub (https://github.com/STUNeilChen/Source-data-of-Loxl2deta13-mice-iScience).

## STAR★Methods

### Key resources table

**Table:** 

REAGENT or RESOURCE	SOURCE	IDENTIFIER
**Antibodies**		

DYKDDDDK Tag Polyclonal antibody	Sigma-Aldrich	Cat#F7425; RRID: AB_439687

**Chemicals, peptides, and recombinant proteins**		

HCS(high-content screening)LipidTOX™ Green neutral Lipid Stains	Invitrogen™	H34475

**Critical commercial assays**		

MesenCult™ Adipogenic Differentiation Medium (Mouse)	STEMCELL	Cat#05507

**Deposited data**		

Source data	This paper	Github: https://github.com/STUNeilChen/Source-data-of-Loxl2deta13-mice-iScience
Mouse reference genome build, GRCm39	Genome Reference Consortium	NCBI: https://www.ncbi.nlm.nih.gov/grc/mouse

**Experimental models: Organisms/strains**		

Mouse: C57BL/J,overexpressed L2Δ13	Shanghai Southern Model Animal Co., Ltd	N/A

**Oligonucleotides**		

Primers for adipogenic-related genes, see Table S2	This paper	N/A

**Software and algorithms**		

R version 3.6.3	R Software Foundation	https://www.r-project.org/
MetaboAnalyst 5.0	Pang et al., 2021	https://www.metaboanalyst.ca/
MicrobiomeAnalyst	Dhariwal et al., 2017	https://www.microbiomeanalyst.ca/
QIIME2	Bolyen et al., 2019	https://qiime2.org/

### Resource availability

#### Lead contact

Further information and requests for resources and reagents should be directed to and will be fulfilled by the lead contact, Li-Yan Xu (lyxu@stu.edu.cn).

#### Materials availability

This study did not generate new unique reagents

### Experimental model and subject details

#### Ethics statement

Animal experiments were approved by the Animal Ethics Review of the Animal Management Center of Shantou University (Ethics Review Number: SUMC2019-404). The Guangdong Provincial Animal Test Certificate was applied to all animal experiments (No.00258438).

#### Animals

We commissioned Shanghai Southern Model Animal Co., Ltd. to generate the L2Δ13 mice used in this study. GT(ROSA)26SOR (ENSMUSG00000086429) is the gene name of the insertion site (Rosa26), which is located on chromosome 6 (113,076,031). The model was constructed via homologous recombination and the embryonic stem cell-targeting method, and resulted in insertion of a CAG promoter-loxP-Neo-loxP-loxL2Δ13-polyA gene at the Rosa26 locus. The loxP-pGK-Neo-polyA-loxP expression frame prevents translation of the downstream target gene L2Δ13. Utilizing the Cre-loxP principle, mice with high conditional expression of the target gene L2Δ13 were mated with Cre-expressing mice (EIIA-Cre), and the loxP-pGK-neo-polyA-loxP expression frame was knocked out in the offspring of double-positive mice. The CAG promoter drives high expression of the L2Δ13 gene in all organs. The L2Δ13 mice used in this study were homozygous (Figure S1). Notably, high expression of the L2Δ13 gene in mice corresponds to the human LOXL2 clipped subtype L2Δ13 identified in our previous research(Lv et al., 2014). All mice were housed in the Laboratory Animal Center of Shantou University Medical College, under conditions of 20–23°C and a humidity of 40–50%. The laboratory environment was maintained on a 12-hour day/night cycle, and ultraviolet radiation was administered twice a day for 1 h. To maintain the pathogen-free status of the experimental animals, all drinking water was filtered and the food and drinking water were sterilized.

#### Study design

Four groups of mice were used in this study. A list of samples is provided in Table S1. The first group consisted of fifteen 4-week-old male, specific pathogen-free (SPF) grade C57BL/J wild-type mice (WT, n = 8) and overexpressing L2Δ13 homozygous mice (L2Δ13, n = 7). All mice were fed the same regular diet (RD). Weight monitoring began in week 4 and continued until week 16. Diet and water were monitored from weeks 6–16. Fecal samples were collected in weeks 8 and 16 and subsequently used for 16S rDNA gene sequencing.

A high-fat diet mouse model was constructed to validate the phenotype of the L2Δ13 mice. This group included nine WT and seven L2Δ13 mice (total, n = 16). Mice in both groups were fed by their mothers from birth until weaning, after which they were fed a regular diet (RD) or a high-fat diet (HFD) containing 60% fat at 4 weeks. The experiment ended when the mice were 20-weeks old. Growth of the mice was evaluated weekly. Fecal samples were collected on weeks 4, 8, 12, 16, and 20 and used for 16S rDNA gene and metabolome sequencing. The body weight of the mice, and food and water intake were recorded once a week. At the end of week 20, eyeballs were collected for blood sampling, and the mice were euthanized. The omental fat (mWAT), inguinal fat (iWAT), and perigonadal fat (pgWAT) of mice were measured and documented. Some tissues were frozen at −80°C, while others were fixed with 4% paraformaldehyde and used to prepare paraffin sections for later use.

The third group of mice was used to investigate the mechanism by which L2Δ13 suppresses the development of obesity in mice. Mice in this group (n_WT_ = 6, n_L2Δ13_ = 6) were fed under the same conditions as those described for the second group in the high-fat mouse model. Plasma tissues were collected for metabolome sequencing, and iWAT tissues were collected for transcriptome and metabolome sequencing on week 20.

A fourth group consisted of twenty 4-week-old female, specific pathogen-free (SPF) grade C57BL/J wild-type mice (WT, n = 10) and overexpressing L2Δ13 homozygous mice (L2Δ13, n = 10). All mice were fed the same regular diet (RD) between weeks 4–17, and then fed a high-fat diet (HFD) containing 60% fat from the 18th week to 24th week. The experiment ended when the mice were 24 weeks old. Weight monitoring began in week 4 and continued until week 24. Fecal samples were collected in weeks 8, 16, 20 and 24 and subsequently used for 16S rDNA gene sequencing.

### Method details

#### High-throughput 16S rRNA gene amplicon sequencing

Microbial DNA was extracted and amplified as previously described (Taguer et al., 2021; Wang et al., 2021). The primer sequences specific for the V3–V4 region were: 341F 5′-CCTAYGGGRBGCASCAG-3′ and 806R 5′-GGACTACNNGGGTATCTAAT-3′. Sequence data was analyzed using QIIME2 software(Bolyen et al., 2019). FastQC and Trimmomatic were used to trim and align the paired-end reads, and dada2 (Qiime2) was used to construct the feature table (Callahan et al., 2016). Taxonomic alignment was performed using the pretrained Naive Bayes classifier silva-138-99-nb-classifier in Qiime2.

Downstream 16S rRNA analysis was performed using the *MicrobiomeAnalystR* R package (Dhariwal et al., 2017). First, rarefaction curves were used to assess sequencing depth (Figure S2). Next, the relative abundance of each group and sample was computed based on the normalized operational taxonomic units (OTUs). The Shannon index was calculated based on the richness of the phyla, classes, orders, and families to characterize alpha diversity. The diversity between groups was assessed by beta-diversity analysis, which was calculated using principal coordinate analysis (PCoA) and the Bray Curtis index. An R value near 1 suggests that these groups were dissimilar, whereas a value near 0 implies no significant dissimilarity. Next, four differential analyses (t-test, DESeq2, LEfSe (Segata et al., 2011), and random forest) were performed to filter abnormal bacteria between L2Δ13 and WT mice. PICRUSt2 was used to predict the functional content of the metagenomes and the potential microbial biological functions (Douglas et al., 2020). The PICRUSt2 results were visualized via the *ggplot2* R package.

#### Metabolomic analysis

##### Fecal metabolites

A fecal sample (100 mg) was added to 100 μL of pure water. The solution was centrifuged at 16,000 g for 15 min at 4°C and 800–850 μL of the supernatant was transferred into a new tube. Concentrated samples were dried in a vacuum and then added to 40 μL of 15 mg/mL methoxyamine pyridine, which was then vortexed for 30 s, and incubated at 37°C for 90 min. Next, 40 μL of BSTFA reagent (containing 1% TMCS) was added to the mixture and incubated at 70°C for 60 min. The mixture was centrifuged at 12,000 rpm for 5 min and the supernatant was transferred to an injection bottle (Dunn et al., 2011). Twenty microliters of each sample was used for quality control (QC).

To improve metabolite coverage, non-targeted metabolomics analysis was performed using liquid chromatography with tandem mass spectrometry (LC-MS/MS), and a Q Exactive high-resolution mass spectrometer (Thermo Fisher Scientific, USA) was used to acquire data in positive and negative ion modes. LC-MS/MS data was processed by Compound Discoverer 2.1 and included intelligent peak extraction, metabolite identification, and peak alignment.

##### Lipid metabolites in plasma and adipose tissue

Lipid metabolites were detected in plasma and WAT samples from L2Δ13 and WT mice, as described previously (Dunn et al., 2011). A Q Exactive high-resolution mass spectrometer (Thermo Fisher Scientific, USA) was used to collect data for non-targeted lipidomics analysis (LC-MS/MS). Intelligent peak extraction, lipid identification, and peak alignment were performed using LipidSearch 4.1.

#### RNA sequencing of WAT

To investigate the mechanism through which L2Δ13 inhibited obesity in mice, we evaluated gene expression in iWAT from 20-week-old mice fed the HFD, and 8-week-old mice fed the RD. FastQC was used to reduce the raw readings in FASTQ files following the extraction and sequencing of total RNA. Then, using STAR software and *Mus musculus*.GRCm38.89.gtf as a reference, whole-transcriptome reads were aligned. The fragments per kilobase of transcript per million mapped reads (FPKM) method was used to normalize the gene counts. Differential expression analyses were performed using the R package *DESeq2*. The threshold for differentially-expressed genes (DE-Gs) was an adjusted p value < 0.05, and absolute log2fold-change > 0.585. DEG enrichment was analyzed using the Metascape (http://metascape.org) webserver. Potential functions with an adjusted p value < 0.05 were selected.

#### Hematoxylin-eosin (HE) staining

Adipose tissue was fixed in 4% paraformaldehyde. Tissues embedded in paraffin were cut into 4 μm thick sections and then subjected to deparaffinization, rehydration, and hematoxylin and eosin (H&E) staining. At least six mouse tissues per group were used for the experiment.

#### Adipogenic differentiation of primary stromal vascular fraction cells

Primary stromal vascular fraction (SVF) cells were obtained from the iWAT of 8-week-old mice, and isolated SVF cells were cultured at 37°C in a 5% CO_2_ incubator (Aune et al., 2013). Cells were stimulated with adipogenic differentiation medium [MesenCult Adipogenic Medium (STEMCELL Technologies)] upon reaching confluence (Day 0) (Patra et al., 2020). Cells were incubated at 37°C under normoxic conditions and the medium was changed every 3 days until the cells had matured to form adipocytes (Day 8). On Day 8, adipogenic differentiation was assessed by staining lipid droplets with oil red O.

#### Western blotting

Following cell collection, western blot analysis was performed. Extracts were prepared from cells using Laemmli sample buffer (Bio-Rad, USA). Cell lysates were fractionated by 8% SDS-polyacrylamide gel electrophoresis and transferred to polyvinylidene fluoride membranes. The membranes were blocked with 5% non-fat dry milk in Tris-buffered saline Tween 20 for 1 h at 25°C, then incubated overnight at 4°C with antibodies against Flag (Proteintech, 20543-1-AP, 1:2000) and β-actin (1:1000; Santa Cruz Biotechnology, sc-47778). After 16 h, the membranes were incubated for 2 h at room temperature with 1:2000 HRP-conjugated secondary antibodies after three 5 min washes. Western Blotting Luminol Reagent (Santa Cruz, sc-2048) was used to detect the signals, which were then visualized using a Bio-Rad ChemiDoc MP fluorescent imaging system.

#### Oil red O and HCS LipidTOX neutral lipid staining

Oil red O (Solarbio, G1260) or neutral lipid droplet staining was performed to confirm adipogenic differentiation. Samples were fixed in 4% paraformaldehyde for 30 min. For oil red O staining, samples were treated with a solution of oil red O and water (2:3) for an additional 30 min before rinsing twice with water. Images were captured under brightfield using a Zeiss microscope. To assess the size of adipogenic cells, the incorporated oil red O dye was extracted in a culture dish with 1 mL isopropanol by shaking for 5 min at 25°C. One hundred microliters of the medium were collected and the absorbance was read using a multi-scan spectrum at 492 nm. LipidTOX Green neutral lipid stain (Molecular probes, H34475, 1:200) was added to the samples for 45 min to stain neutral lipid droplets and images were captured using a Zeiss LSM 800 laser confocal microscope. Quantitative analysis of the lipid droplets was performed using ImageJ, which was used to measure the diameters of the lipid droplets in each image. The proportion of lipid droplets in each diameter was then estimated.

#### qRT-PCR

Total RNA was purified from iWAT using TRIzol (Life Technology, USA), and then reverse-transcribed to form cDNA using HiScript III-RT Super-Mix for qPCR (+gDNA wiper) (Vazyme, Nanjing, China, R323-01), according to the manufacturer’s instructions. Markers of lipid metabolism were analyzed by quantitative RT-PCR using ChamQ Universal SYBR qPCR Master Mix (Vazyme, Nanjing, China, Q711-02) and a QuantStudio 5 Real-Time PCR System (Thermo Fisher, USA) according to the manufacturer’s protocol. Relative expression was determined using the comparative delta-delta Ct (2-ΔΔCT) method, as previously described (Livak and Schmittgen, 2001). The primer sequences used in this experiment are listed in Table S2.

### Quantification and statistical analysis

*MetaboAnalystR* R package and MetaboAnalyst 5.0 were used for metabolomic analyses (Pang et al., 2021). The Wilcoxon rank-sum test, fold-change analysis, PCA, and partial least-squares discriminant analysis (PLS-DA) were used to select differentially-expressed metabolites (DE-Ms). Metabolites with an adjusted p < 0.05, absolute log2 fold-change > 0.585 (fecal) or 0.263 (WAT), and VIP ≥1 were considered as DE-Ms. Correlations between body weight and microbiota, body weight and metabolites, and microbiota and metabolites were assessed using Spearman’s correlation. The results were visualized using the *ggplot2* and *corrplot* R packages, and the criterion was set to an adjusted p < 0.05. GraphPad Prism 7.0, was used for statistical analyses. Multiple t-tests were used to compare L2Δ13 mice with WT mice, and a p < 0.05 was considered to indicate significance.

## Data Availability

• The source data is available at Github and is listed in the Key resources table. • This paper does not report any original code. • Any additional information required to reanalyze the data reported in this paper is available from the Lead contact upon request.

## Supplemental information

Document S1. Figures S1–S7 and Table S2

Table S1. Four cohorts of mice which used in the study, related to STAR Methods

Table S3. Differentially-expressed metabolites in plasma at 20 weeks, related to Figure 6
