# Supplementary material for: L2Δ13, a splicing isoform of lysyl oxidase-like 2, causes adipose tissue loss via the gut microbiota and lipid metabolism
Source: iScience. 2022 Aug 15;25(9):104894. doi: 10.1016/j.isci.2022.104894 (PMC9436769; doi:10.1016/j.isci.2022.104894)
Supplement: Document S1. Figures S1–S7 and Table S2 [file mmc1.pdf]

iScience, Volume *25*

Supplemental information

L2Δ13, a splicing isoform of lysyl oxidase-like 2, causes adipose tissue loss via the gut microbiota and lipid metabolism

Yang Chen, Li-Xia He, Jin-Ling Chen, Xin Xu, Juan-Juan Wang, Xiu-Hui Zhan, Ji-Wei Jiao, Geng Dong, En-Min Li, and Li-Yan Xu

Supplementary Materials

Supplementary figures

Figure S1. Expression of LOXL2Δ13 in various mouse tissues. Related to STAR Methods.

Figure S2. Rarefaction curve analysis of 16S rRNA sequencing. Related to STAR Methods.

Figure S3. 16S rRNA sequencing for female mice under RD. Related to Figure 1.

Figure S4. 16S rRNA sequencing for female mice under HFD. Related to Figure 3.

Figure S5. PICRUSt2 analysis of microbiota under HFD. Related to Figure 4.

Figure S6. Correlation analysis between DEMs and 22 crucial gut microbial species. Related to Figure 5.

Figure S7. In vitro culture of primary adipose stem cell SVF. Related to STAR Methods.

Supplementary figures

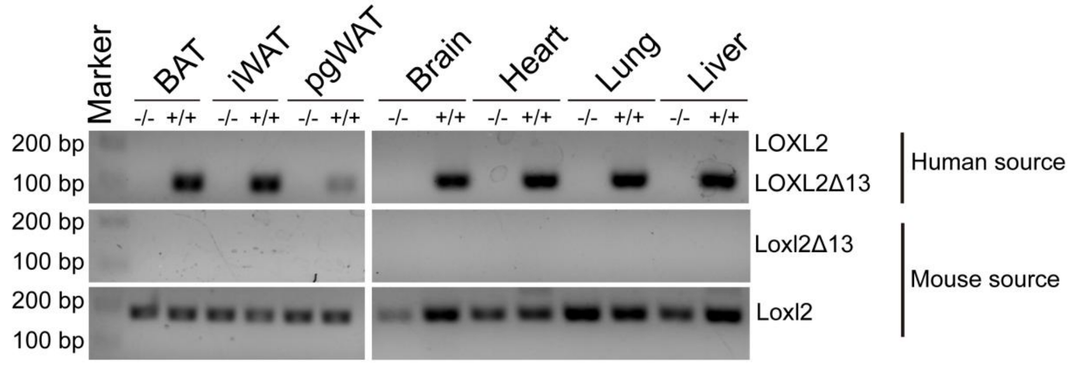

Figure S1. Expression of LOXL2Δ13 in various mouse tissues. Related to STAR Methods. RT-PCR was used to detect the expression of LOXL2Δ13 in various mouse tissues, such as brain, heart, lung, liver, scapular (BAT), inguinal (iWAT) and perigonadal (pgWAT) adipose tissue samples. -/-: control mice; +/+: LOXL2Δ13 transgenic mice.

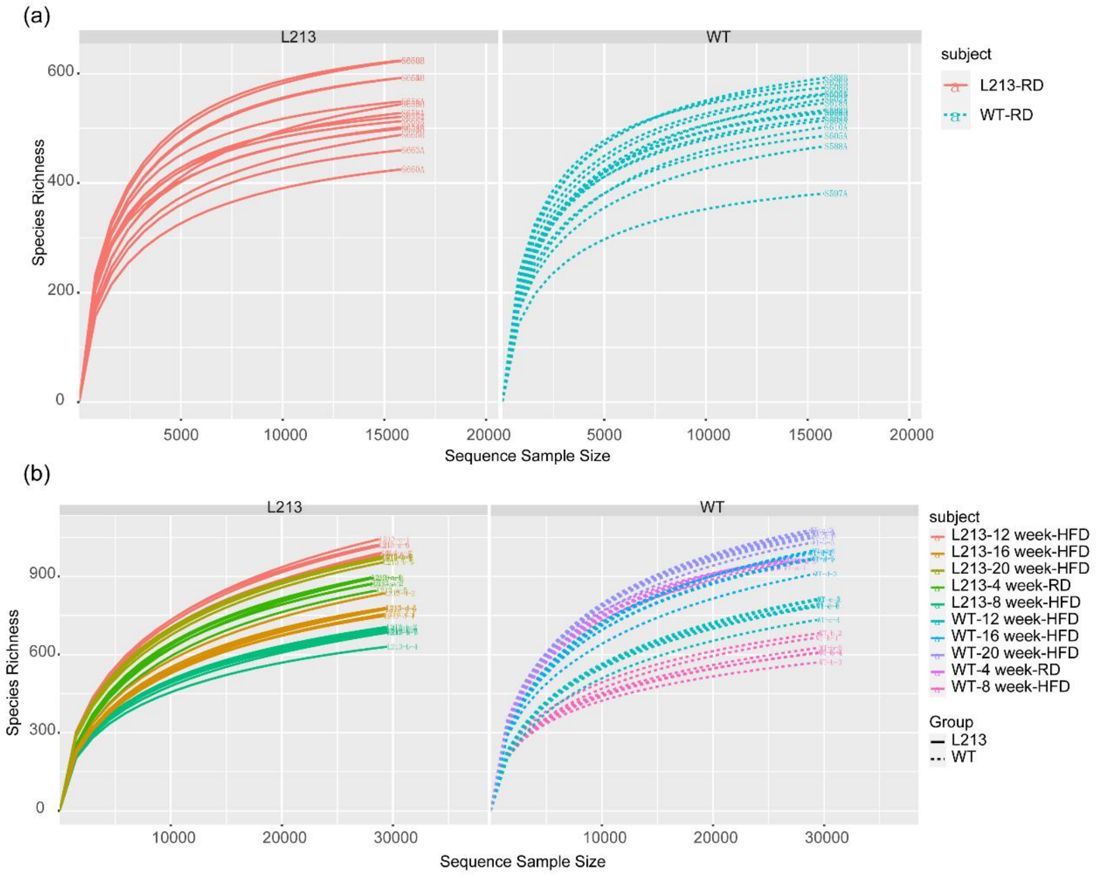

Figure S2. Rarefaction curve analysis of 16S rRNA sequencing. Related to STAR Methods. Rarefaction curves were used to evaluate the sequencing depth. The upper part is the 16S rRNA analysis under a regular diet, while the bottom part represents the high-fat diet.

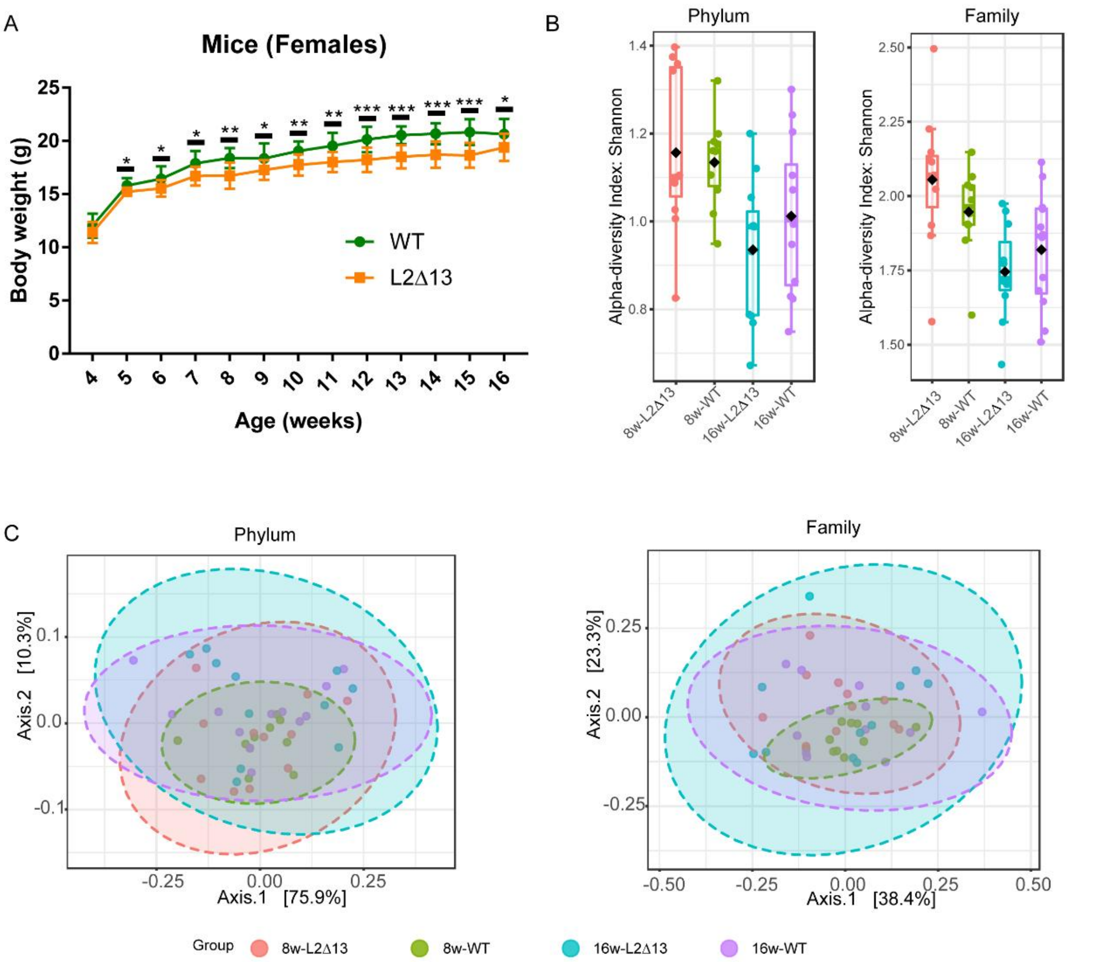

Figure S3. 16S rRNA sequencing for female mice under RD. Related to Figure 1.
A. Body weight of female mice. **B.** Alpha diversity of the microbiome in female mice. **C.** Beta diversity of the microbiome in female mice.

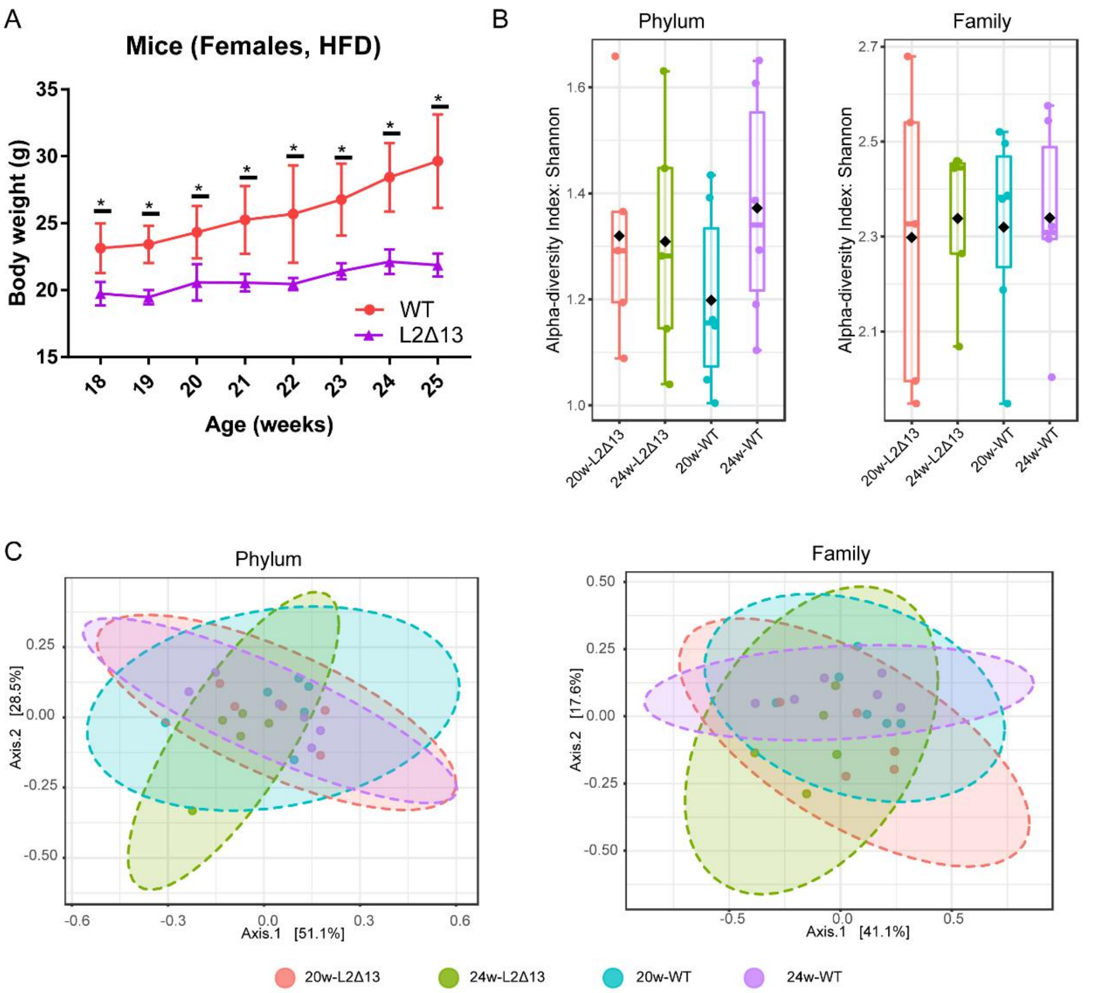

Figure S4. 16S rRNA sequencing for female mice under HFD. Related to Figure 3. A. Body weight of female mice under HFD. **B.** Alpha diversity of the microbiome in female mice under HFD. **C.** Beta diversity of the microbiome in female mice under HFD.

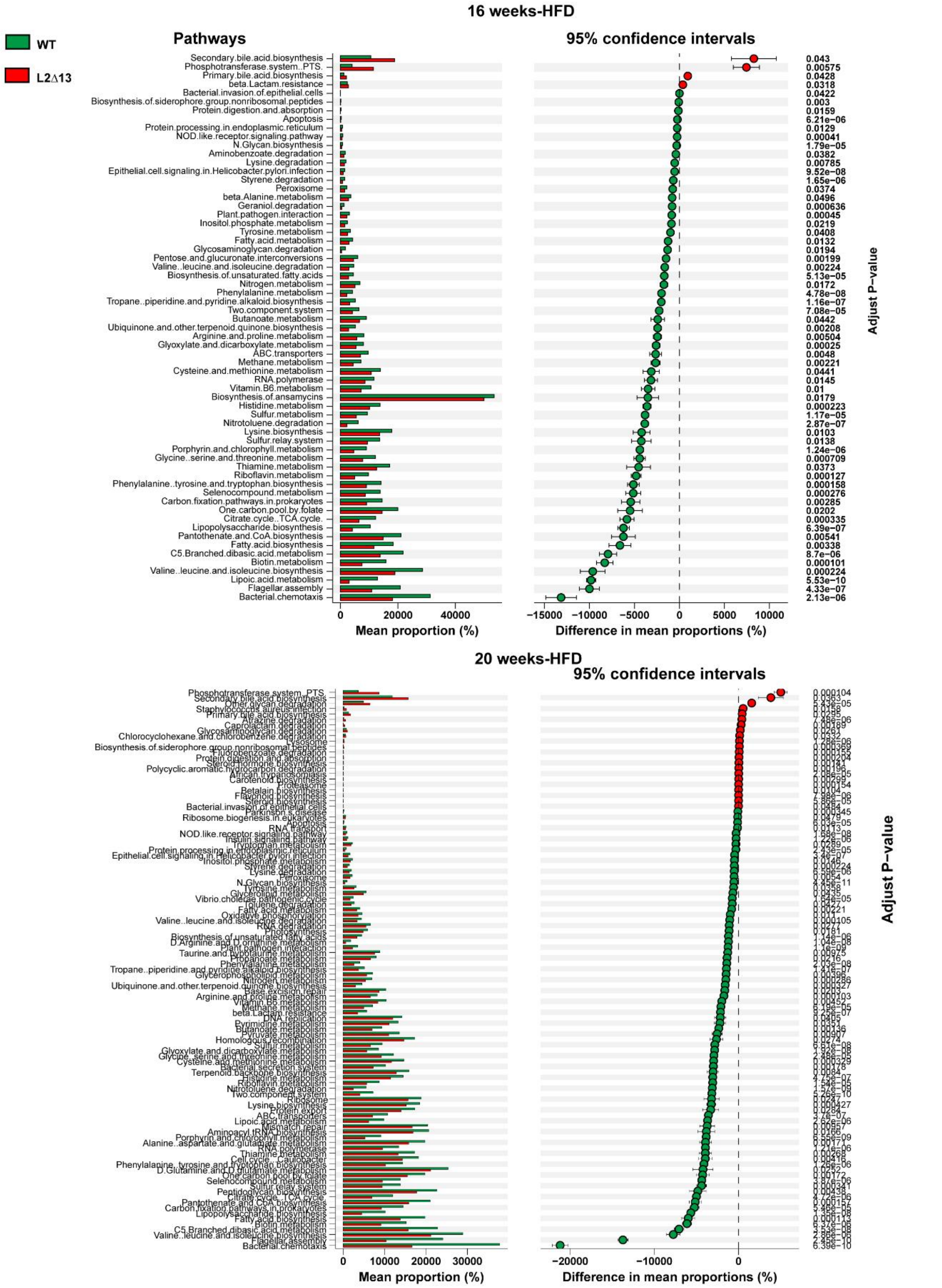

Figure S5. PICRUSt2 analysis of the microbiotas under HFD. Related to Figure 4.

The upper part is the PICRUSt2 results at 16 weeks, and the bottom part shows the results at 20 weeks.

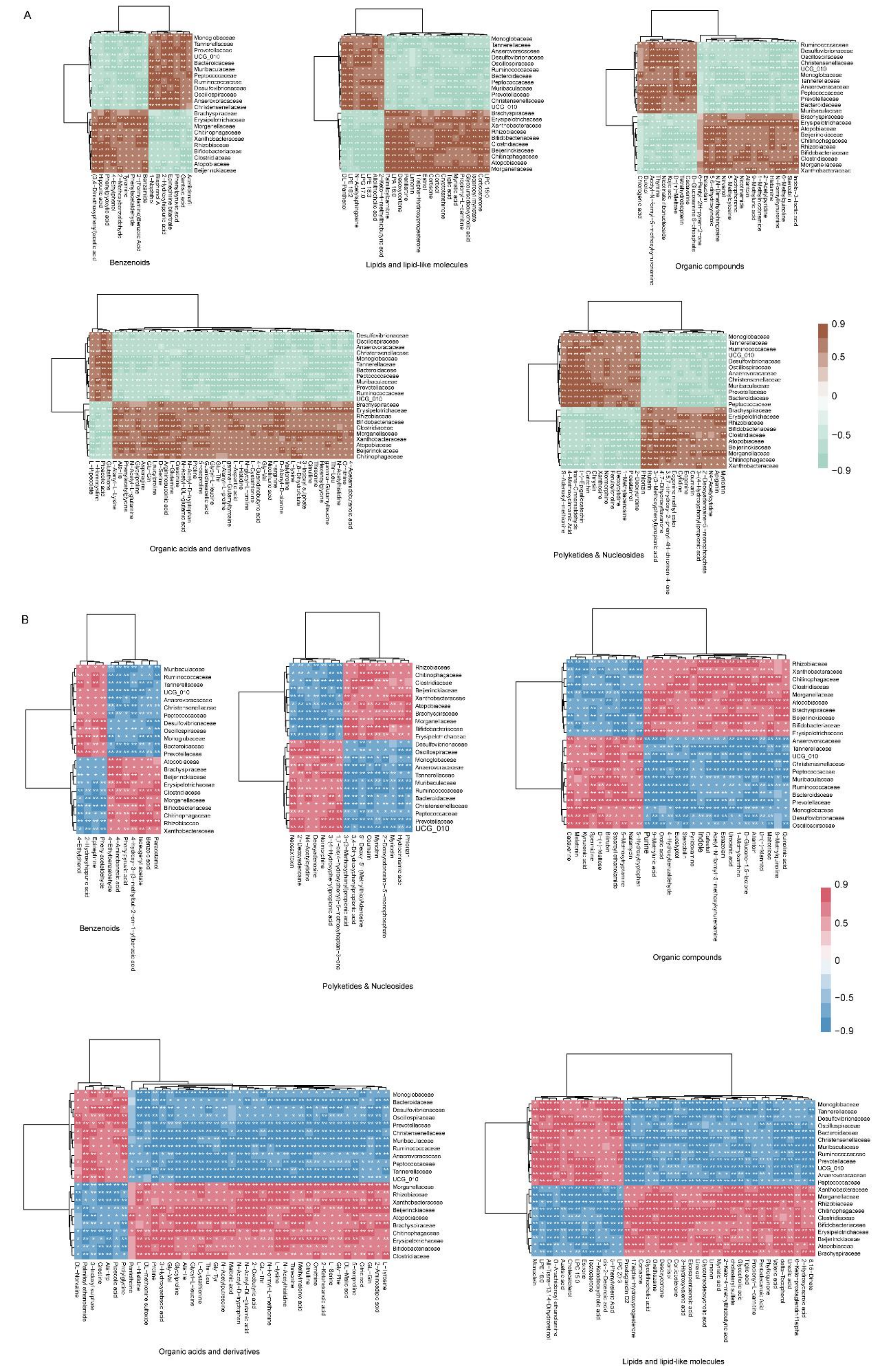
A
Benzenoids
Lipids and lipid-like molecules
Organic compounds
Organic acids and derivatives
Polyketides & Nucleosides
B
Benzenoids
Polyketides & Nucleosides
Organic compounds
Organic acids and derivatives
Lipids and lipid-like molecules

Figure S6. Correlation analysis between DEMs and 22 crucial gut microbial species. Related to Figure 5. (A) DEMs were divided into five subclasses, and the heatmap shows the Spearman correlation coefficient and p-value between DEMs and microbiotas at 16 weeks. **(B)** Spearman correlation analysis between DEMs and 22 crucial gut microbial species at 20 weeks.

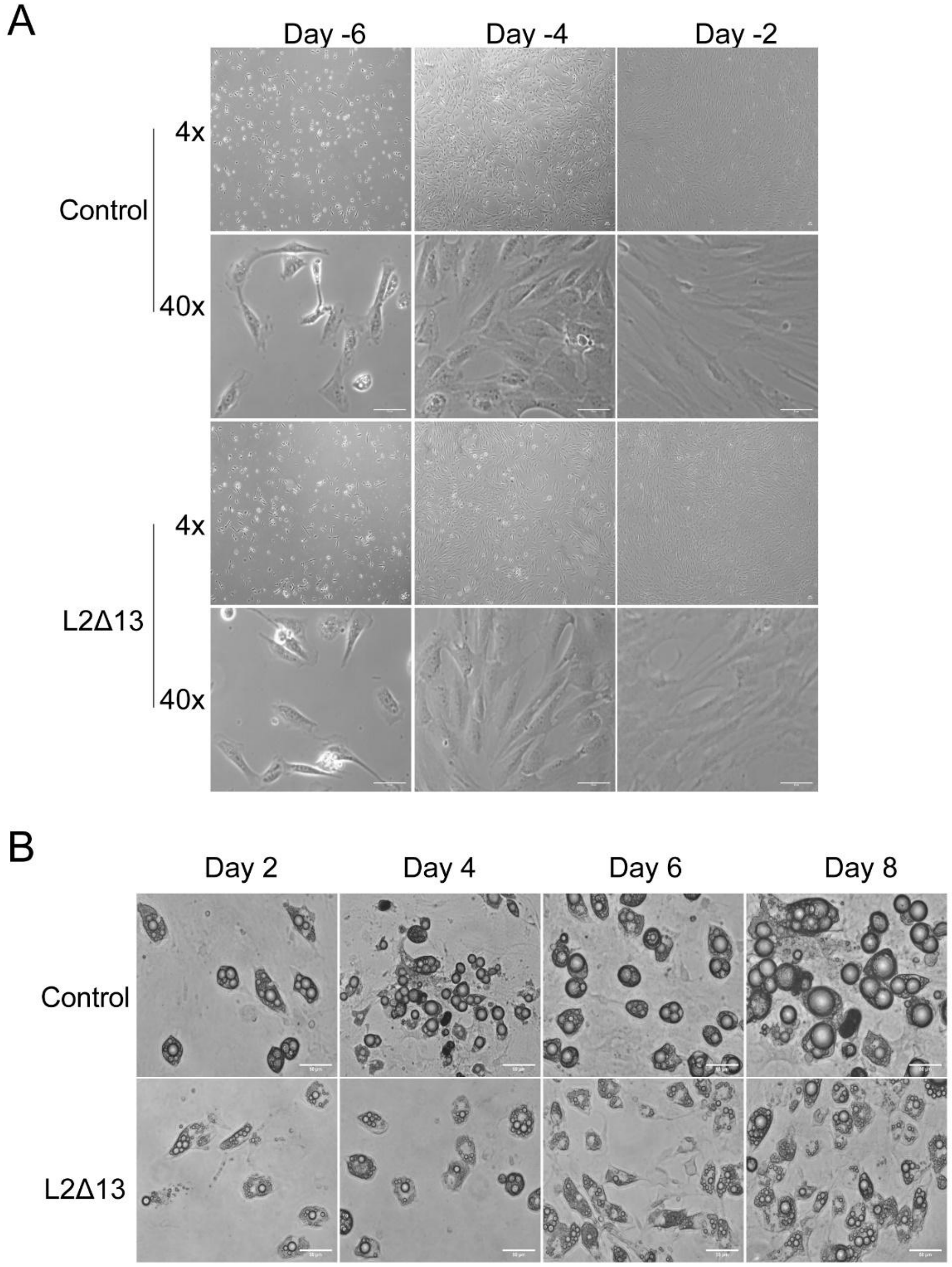

Figure S7. In vitro culture of primary adipose stem cell SVF. Related to STAR Methods. (A) After inoculation, cells in the P0 generation of SVFs began to grow adhering to the wall. Continuous observation under the microscope showed that the proliferation and growth of the cells were slow in the first 2 days (Day -6) and began to grow exponentially on the 4th day (Day -4). On the 6th day, the cells reached the

contact inhibition state and appeared as fibroblasts (Day -2). **(B)** The cells grew to more than 90% fusion (Day 0), at which time adipogenic differentiation could be induced in vitro. After 2 days of adipogenic culture, a few cells became round and lipid droplets appeared (Day 2). With the increase in time, the lipid droplets also increased and gathered into larger droplets, and mature adipocytes were formed after 8 days. Scale bars represent 50μm.

Supplementary tables

Table S2. Primers for the qRT-PCR assays. Related to Figure 8 and Figure S1.

Table S4. Enrichment analysis of DEGs between L2Δ13 and WT mice under the high-fat diet. Related to Figure 7.

Table S5. Enrichment analysis of DEGs between L2Δ13 and WT mice under the regular diet. Related to Figure 8.

Table S2. The primers for the qRT-PCR assay. Related to Figure 8 and Figure S1.

Primer	Sequence（5′-3′）
Mogat2-F	TGGGAGCGCAGGTTACAGA
Mogat2-R	CAGGTGGCATACAGGACAGA
Scd1-F	TTCTTGCGATACACTCTGGTGC
Scd1-R	CGGGATTGAATGTTCTTGTCGT
Pck1-F	AGCATTCAACGCCAGGTTC
Pck1-R	CGAGTCTGTCAGTTCAATACCAA
Mttp-F	ATACAAGCTCACGTACTCCACT
Mttp-R	TCTCTGTTGACCCGCATTTTC
Angptl4-F	CCCCACGCACCTAGACAATG
Angptl4-R	GCCTCCATCTGAAGTCATCTCA
Nr1d1-F	TACATTGGCTCTAGTGGCTCC
Nr1d1-R	CAGTAGGTGATGGTGGGAAGTA
Mest-F	CTCCAGAACCGCAGAATCAAC
Mest-R	AGATACCTCCATTCGACAGACAG
Ttpa-F	GGGCCATTGGTTGGGAAACA
Ttpa-R	AGCAGGAAAGCGTCTGTGAG
Oxtr-F	GATCACGCTCGCCGTCTAC
Oxtr-R	CCGTCTTGAGTCGCAGATTC
Npr3-F	GTCTACAGCGACGACAAACTC
Npr3-R	AGGTCCAAGTCTTTGGTCTCG
Pdk4-F	GACAGACGCTATCATCTACTT
Pdk4-R	CTTGGGATACACCAGTCAT
Adipoq-F	GTTCCCAATGTACCCATTCGC
Adipoq-R	TGTTGCAGTAGAACTTGCCAG
Plin4-F	TGTGACCAGCAGTGAAGATG
Plin4-R	TCCGACAAGGGCCGACT
Pparg-F	GTTGATTTCTCCAGCATTTC
Pparg-R	TTGATCGCACTTTGGTATT
Pgc-1α-F	ACAGGAACAGCAGCAGAG
Pgc-1α-R	TAAGGTTCGCTCAATAGTC
Foxo1-F	ATGCTCAATCCAGAGGGAGG

Foxo1-R	ACTCGCAGGCCACTTAGAAAA
Apq7-F	AATATGGTGCGAGAGTTTCTGG
Apq7-R	AACCCAAGTTGACACCGAGAT
C/ebpd-F	CCTGCCATGTACGACGACGAG
C/ebpd-R	GCCGCTTTGTGGTTGCTGTTG
β-actin-F	GTGACGTTGACATCCGTAAAGA
β-actin-R	GCCGGACTCATCGTACTCC
**Primers for the RT-PCR assay to identify the alternative splicing L2Δ13 in mice**	
LOXL2&L2Δ13-qF	ACTGACGTGCCCCCTGGAGAC
LOXL2&L2Δ13-qR	CCCAAGCTTCCAGAGCCCGCTGAAGTG
Loxl2Δ13-qF	GGGAGACATTCAGAAGAGTT
Loxl2Δ13-qR	AAGGCTCCACCTGGAACAG
Loxl2-qF	GAGAACAAGGCATCACCA
Loxl2-qR	CATCATAGCGGCTCCTG

Table S4. The enrichment analysis of DEGs between L2Δ13 and WT mice under the high-fat diet. Related to Figure 7.

Description	ID	Enrichment	LogP	count
HALLMARK	hallmark myogenesis	21.48485591	-54.60093701	53
HALLMARK	hallmark hypoxia	8.512867435	-13.07673682	21
HALLMARK	hallmark tnfa signaling via nfkb	6.080619597	-7.492884664	15
HALLMARK	hallmark epithelial mesenchymal transition	4.864495677	-5.102684911	12
HALLMARK	hallmark glycolysis	3.648371758	-3.048849647	9
HALLMARK	hallmark kras signaling up	3.648371758	-3.048849647	9
HALLMARK	hallmark il2 stat5 signaling	3.259293586	-2.465739178	8
HALLMARK	hallmark apical junction	3.242997118	-2.452570907	8
HALLMARK	hallmark kras signaling dn	3.242997118	-2.452570907	8
HALLMARK	hallmark complement	3.242997118	-2.452570907	8
HALLMARK	hallmark xenobiotic metabolism	3.242997118	-2.452570907	8
HALLMARK	hallmark fatty acid metabolism	3.591927188	-2.447822275	7
HALLMARK	hallmark apoptosis	3.524996868	-2.403037325	7
HALLMARK	hallmark cholesterol homeostasis	4.382428538	-1.875913284	4
HALLMARK	hallmark estrogen response early	2.837622478	-1.907040908	7
HALLMARK	hallmark mtorc1 signaling	2.432247839	-1.417319171	6
HALLMARK	hallmark uv response up	2.565662277	-1.331860972	5
HALLMARK	hallmark p53 pathway	2.432247839	-1.417319171	6
KEGG Pathway	Hypertrophic cardiomyopathy	7.61255771	-7.253208204	12
KEGG Pathway	Dilated cardiomyopathy	7.211896778	-6.987206734	12
KEGG Pathway	Oxytocin signaling pathway	5.489825272	-6.966679336	15
KEGG Pathway	Adrenergic signaling in cardiomyocytes	4.948162512	-5.60439587	13
KEGG Pathway	Arrhythmogenic right ventricular cardiomyopathy	6.761153229	-5.14758308	9
KEGG Pathway	Cardiac muscle contraction	5.885998229	-5.107386356	10
KEGG Pathway	p53 signaling pathway	5.55082333	-3.577532587	7
KEGG Pathway	Calcium signaling pathway	3.394789249	-3.359751863	11
KEGG Pathway	HIF-1 signaling pathway	4.114896059	-3.121666065	8
KEGG Pathway	MAPK signaling pathway	2.811455972	-3.085005962	13
KEGG Pathway	Glycolysis / Gluconeogenesis	4.567534626	-2.694775849	6
KEGG Pathway	Biosynthesis of amino acids	4.391860217	-2.608033952	6
KEGG Pathway	Glucagon signaling pathway	3.735133456	-2.555405189	7
KEGG Pathway	Carbon metabolism	3.386943049	-2.320188107	7
KEGG Pathway	Insulin signaling pathway	3.024857368	-2.282726103	8
KEGG Pathway	cGMP-PKG signaling pathway	2.718770611	-2.01360266	8
KEGG Pathway	Tight junction	2.702683211	-1.998994421	8
KEGG Pathway	Gastric acid secretion	3.806278855	-1.99243579	5
KEGG Pathway	Apoptosis	2.875246617	-1.944183877	7
KEGG Pathway	Apelin signaling pathway	2.81450197	-1.896911551	7
KEGG Pathway	Cholinergic synapse	3.031549531	-1.834944668	6
KEGG Pathway	Regulation of lipolysis in adipocytes	4.152304205	-1.80529018	4

KEGG Pathway	Insulin secretion	3.207538361	-1.696595768	5
KEGG Pathway	GnRH signaling pathway	3.102944719	-1.641152078	5
KEGG Pathway	Longevity regulating pathway	3.069579722	-1.623209279	5
KEGG Pathway	human papillomavirus infection	4.391860217	-1.514321113	3
KEGG Pathway	AMPK signaling pathway	2.556455947	-1.511879774	6
KEGG Pathway	Pentose phosphate pathway	4.177623134	-1.458481324	3
KEGG Pathway	Fructose and mannose metabolism	4.177623134	-1.458481324	3
KEGG Pathway	human immunodeficiency virus 1 infection	4.177623134	-1.458481324	3
KEGG Pathway	NF-kappa B signaling pathway	2.771562273	-1.457158253	5
KEGG Pathway	Proteoglycans in cancer	2.114599364	-1.431628761	8
KEGG Pathway	Mineral absorption	3.644309542	-1.309247644	3
KEGG Pathway	Salivary secretion	2.8908447	-1.301222342	4

Table S5. The enrichment analysis of DEGs between L2Δ13 and WT mice under the regular diet. Related to Figure 8.

Category	Description	Enrichment	LogP	Count
Hallmark	HALLMARK INTERFERON GAMMA RESPONSE	4.93919551	-32.3623578	75
Hallmark	HALLMARK ALLOGRAFT REJECTION	4.80748363	-30.62248392	73
Hallmark	HALLMARK IL2 STAT5 SIGNALING	3.97121247	-20.38286784	60
Hallmark	HALLMARK ADIPOGENESIS	3.819644528	-18.81841703	58
Hallmark	HALLMARK INTERFERON ALPHA RESPONSE	5.024061412	-16.59416339	37
Hallmark	HALLMARK EPITHELIAL MESENCHYMAL TRANSITION	3.490364827	-15.39977016	53
Hallmark	HALLMARK MYOGENESIS	3.424508887	-14.74903235	52
Hallmark	HALLMARK INFLAMMATORY RESPONSE	3.292797007	-13.48152311	50
Hallmark	HALLMARK TNFA SIGNALING VIA NFKB	3.095229186	-11.6672277	47
Hallmark	HALLMARK APICAL JUNCTION	3.029373246	-11.08624397	46
Hallmark	HALLMARK KRAS SIGNALING UP	3.029373246	-11.08624397	46
Hallmark	HALLMARK IL6 JAK STAT3 SIGNALING	3.784824145	-8.424015242	25
Hallmark	HALLMARK FATTY ACID METABOLISM	2.834306284	-7.604939401	34
Hallmark	HALLMARK ESTROGEN RESPONSE LATE	2.568381665	-7.369752571	39
Hallmark	HALLMARK ESTROGEN RESPONSE EARLY	2.502525725	-6.891206902	38
Hallmark	HALLMARK APOPTOSIS	2.617875881	-6.365660197	32
Hallmark	HALLMARK G2M CHECKPOINT	2.370813845	-5.975586868	36
Hallmark	HALLMARK E2F TARGETS	2.370813845	-5.975586868	36
Hallmark	HALLMARK COMPLEMENT	2.304957905	-5.539004196	35
Hallmark	HALLMARK XENOBIOTIC METABOLISM	2.304957905	-5.539004196	35
Hallmark	HALLMARK KRAS SIGNALING DN	2.239101964	-5.116918936	34
Hallmark	HALLMARK TGF BETA SIGNALING	3.658663341	-5.111827107	15
Hallmark	HALLMARK UV RESPONSE DN	2.469597755	-4.98321479	27
Hallmark	HALLMARK MITOTIC SPINDLE	2.117979984	-4.360772374	32
Hallmark	HALLMARK HYPOXIA	2.107390084	-4.317345454	32
Hallmark	HALLMARK P53 PATHWAY	2.041534144	-3.94043814	31
Hallmark	HALLMARK APICAL SURFACE	3.292797007	-3.460901635	11
Hallmark	HALLMARK UV RESPONSE UP	2.084048738	-3.436797578	25
Hallmark	HALLMARK WNT BETA CATENIN SIGNALING	2.822397434	-2.431099032	9
Hallmark	HALLMARK COAGULATION	1.90886783	-2.410553361	20
Hallmark	HALLMARK HEDGEHOG SIGNALING	2.926930672	-2.316574277	8
Hallmark	HALLMARK MTORC1 SIGNALING	1.712254443	-2.297291006	26
Hallmark	HALLMARK HEME METABOLISM	1.712254443	-2.297291006	26
Hallmark	HALLMARK NOTCH SIGNALING	2.881197381	-2.047101324	7
Hallmark	HALLMARK CHOLESTEROL HOMEOSTASIS	2.135868329	-2.021037727	12
Hallmark	HALLMARK PEROXISOME	1.899690581	-1.924620693	15
Hallmark	HALLMARK ANGIOGENESIS	2.561064338	-1.767473404	7
KEGG Pathway	Cytokine-cytokine receptor interaction	3.268405918	-17.64444471	67
KEGG Pathway	Rap1 signaling pathway	3.324156978	-14.43233417	53

KEGG Pathway	Primary immunodeficiency	7.47553915	-13.95921619	21
KEGG Pathway	Chemokine signaling pathway	3.473719919	-13.92766424	48
KEGG Pathway	Rap1 signaling pathway	3.119491901	-13.44756609	54
KEGG Pathway	T cell receptor signaling pathway	4.219895193	-12.35779793	33
KEGG Pathway	NF-kappa B signaling pathway	4.159322535	-11.11795244	30
KEGG Pathway	Pathways in cancer	2.367479367	-11.07505611	71
KEGG Pathway	PI3K-Akt signaling pathway	2.391610457	-10.98979199	69
KEGG Pathway	B cell receptor signaling pathway	4.637742263	-10.54291963	25
KEGG Pathway	Arrhythmogenic right ventricular cardiomyopathy (ARVC)	4.573329176	-10.39008588	25
KEGG Pathway	NF-kappa B signaling pathway	3.815951671	-10.37927308	31
KEGG Pathway	Leukocyte transendothelial migration	3.697175586	-10.2922253	32
KEGG Pathway	Hematopoietic cell lineage	3.937777864	-10.12042993	29
KEGG Pathway	Valine, leucine and isoleucine degradation	5.487995011	-10.1072038	20
KEGG Pathway	Cell adhesion molecules (CAMs)	3.292797007	-9.908637847	36
KEGG Pathway	PI3K-Akt signaling pathway	2.349246987	-9.47384343	61
KEGG Pathway	Focal adhesion	2.779848729	-8.95526586	42
KEGG Pathway	Regulation of actin cytoskeleton	2.671514552	-8.592246633	43
KEGG Pathway	ECM-receptor interaction	3.854981861	-8.289724408	24
KEGG Pathway	Adherens junction	4.024529675	-8.037799752	22
KEGG Pathway	Axon guidance	2.784765468	-7.995705928	37
KEGG Pathway	Th17 cell differentiation	3.292797007	-7.863537677	28
KEGG Pathway	Tight junction	2.789192758	-7.817739319	36
KEGG Pathway	Th17 cell differentiation	3.32357081	-7.699945117	27
KEGG Pathway	Regulation of lipolysis in adipocytes	4.314699526	-7.581326307	19
KEGG Pathway	Fc gamma R-mediated phagocytosis	3.473719919	-7.316980505	24
KEGG Pathway	Ras signaling pathway	2.33928436	-7.260296386	46
KEGG Pathway	Oxytocin signaling pathway	2.77288169	-6.964690408	32
KEGG Pathway	Oxytocin signaling pathway	2.699684502	-6.870449207	33
KEGG Pathway	Dilated cardiomyopathy	3.365970273	-6.767971349	23
KEGG Pathway	Small cell lung cancer	3.449596864	-6.705102181	22
KEGG Pathway	Th1 and Th2 cell differentiation	3.225597068	-6.656794929	24
KEGG Pathway	Measles	2.850480991	-6.644377674	29
KEGG Pathway	Th1 and Th2 cell differentiation	3.292797007	-6.580833779	23
KEGG Pathway	Calcium signaling pathway	2.532920774	-6.533307088	35
KEGG Pathway	Osteoclast differentiation	2.836871267	-6.393664196	28
KEGG Pathway	Measles	2.584220436	-6.060661646	31
KEGG Pathway	beta-Alanine metabolism	5.098524397	-5.892219947	12
KEGG Pathway	Osteoclast differentiation	2.672414962	-5.840973948	28
KEGG Pathway	NOD-like receptor signaling pathway	2.479282452	-5.820277265	32
KEGG Pathway	Epstein-Barr virus infection	2.070387415	-5.804156899	47
KEGG Pathway	Jak-STAT signaling pathway	2.532920774	-5.697951953	30
KEGG Pathway	Gastric acid secretion	3.336700967	-5.642948446	19
KEGG Pathway	cAMP signaling pathway	2.328240308	-5.641620867	35
KEGG Pathway	Gastric acid secretion	3.292797007	-5.549865141	19

KEGG Pathway	MAPK signaling pathway	2.117720428	-5.397942302	41
KEGG Pathway	HTLV-I infection	2.109448082	-5.35470175	41
KEGG Pathway	Platelet activation	2.634237605	-5.350811772	26
KEGG Pathway	Tryptophan metabolism	4.280636109	-5.337272159	13
KEGG Pathway	Cholinergic synapse	2.748769675	-5.323212344	24
KEGG Pathway	PPAR signaling pathway	3.292797007	-5.291141548	18
KEGG Pathway	Viral myocarditis	3.5718476	-5.263034423	16
KEGG Pathway	Tyrosine metabolism	4.515835895	-5.244734182	12
KEGG Pathway	aldosterone synthesis and secretion	2.840844476	-5.176498072	22
KEGG Pathway	Natural killer cell mediated cytotoxicity	2.574818712	-5.16156185	26
KEGG Pathway	Hippo signaling pathway	2.34659097	-5.145704165	31
KEGG Pathway	Salivary secretion	2.926930672	-4.968181113	20
KEGG Pathway	Hypertrophic cardiomyopathy (HCM)	3.015091235	-4.947604251	19
KEGG Pathway	Adipocytokine signaling pathway	3.245075311	-4.940705304	17
KEGG Pathway	Apoptosis	2.481528179	-4.861602632	26
KEGG Pathway	Phenylalanine metabolism	6.19820613	-4.847598402	8
KEGG Pathway	cGMP-PKG signaling pathway	2.343340201	-4.844787246	29
KEGG Pathway	Insulin secretion	2.863301745	-4.817646419	20
KEGG Pathway	TNF signaling pathway	2.683019783	-4.758767971	22
KEGG Pathway	cAMP signaling pathway	2.098065349	-4.731514994	36
KEGG Pathway	drug metabolism	2.589207903	-4.681961488	23
KEGG Pathway	Salivary secretion	2.802380431	-4.672259702	20
KEGG Pathway	Fluid shear stress and atherosclerosis	2.411625977	-4.634653978	26
KEGG Pathway	Amoebiasis	2.743997505	-4.531774464	20
KEGG Pathway	Aldosterone synthesis and secretion	2.891236396	-4.458237014	18
KEGG Pathway	Glutathione metabolism	3.414752451	-4.440518948	14
